# Remodelling of supernumerary leaflet primordia leads to bicuspid aortic valve caused by loss of primary cilia

**DOI:** 10.1093/cvr/cvaf108

**Published:** 2025-06-11

**Authors:** Ahlam Alqahtani, Lorraine Eley, Jake Newton, Kimberley McDonald, Chloe Connolly, Cindy Rodrigues-Cleto, Kristyna Neffeova, Leonor Lopez, Javier Arias, Christopher J Derrick, Mashael Alaradi, Hana Kolesova, Bill Chaudhry, Deborah J Henderson

**Affiliations:** Bioscience Institute, Newcastle University, Centre for Life, Central Parkway, Newcastle upon Tyne, NE1 3BZ, UK; Bioscience Institute, Newcastle University, Centre for Life, Central Parkway, Newcastle upon Tyne, NE1 3BZ, UK; Bioscience Institute, Newcastle University, Centre for Life, Central Parkway, Newcastle upon Tyne, NE1 3BZ, UK; Bioscience Institute, Newcastle University, Centre for Life, Central Parkway, Newcastle upon Tyne, NE1 3BZ, UK; Bioscience Institute, Newcastle University, Centre for Life, Central Parkway, Newcastle upon Tyne, NE1 3BZ, UK; Bioscience Institute, Newcastle University, Centre for Life, Central Parkway, Newcastle upon Tyne, NE1 3BZ, UK; Institute of Anatomy, First Faculty of Medicine, Charles University, U Nemocnice 3, Prague 2 12 800, Czech Republic; Bioscience Institute, Newcastle University, Centre for Life, Central Parkway, Newcastle upon Tyne, NE1 3BZ, UK; Bioscience Institute, Newcastle University, Centre for Life, Central Parkway, Newcastle upon Tyne, NE1 3BZ, UK; Bioscience Institute, Newcastle University, Centre for Life, Central Parkway, Newcastle upon Tyne, NE1 3BZ, UK; Bioscience Institute, Newcastle University, Centre for Life, Central Parkway, Newcastle upon Tyne, NE1 3BZ, UK; Institute of Anatomy, First Faculty of Medicine, Charles University, U Nemocnice 3, Prague 2 12 800, Czech Republic; Bioscience Institute, Newcastle University, Centre for Life, Central Parkway, Newcastle upon Tyne, NE1 3BZ, UK; Bioscience Institute, Newcastle University, Centre for Life, Central Parkway, Newcastle upon Tyne, NE1 3BZ, UK

**Keywords:** bicuspid aortic valve, second heart field, intercalated valve swelling, Cilia, Aortic valve, Ift88

## Abstract

**Aims:**

Bicuspid aortic valve (BAV), where two valve leaflets are found instead of the usual three, affects 1–2% of the general population and is associated with significant morbidity and mortality. Despite its frequency, the majority of cases remain unexplained. This is, at least in part, because there are two types of valve leaflet primordia: endocardial cushions and intercalated valve swellings (ICVS). Moreover, multiple progenitors make distinct contributions to the formation of these primordia. Genomic studies in mouse and human have suggested a correlation between BAV and malfunctional primary cilia. However, the precise requirement for cilia during early embryonic valvulogenesis remains unknown.

**Methods and results:**

Here, we disrupted primary cilia by deleting the ciliary gene Ift88 in the main progenitor cells forming the aortic valve using specific Cre drivers: Wnt1-Cre for neural crest cells, Isl1-Cre for second heart field (SHF) cells, Tie2-Cre for endocardial-derived cells, and Tnnt2-Cre for direct-differentiating SHF in the ICVS. Loss of Ift88, and thus primary cilia, from neural crest cells and endocardium did not impact aortic valve formation. However, primary cilia were essential in SHF cells for aortic valve leaflet formation, with over half of Ift88^f/f^;Isl1-Cre mutants presenting with BAV. As the valve leaflets were forming, 50% of the Ift88^f/f^;Isl1-Cre mutants had two small leaflets in the position of the usual posterior leaflet, meaning that at this stage, the aortic valve was quadricuspid, which then remodelled to BAV by E15.5. Mechanistic studies demonstrated premature differentiation of SHF cells as the ICVS formed, leading to the formation of a broadened ICVS that formed two posterior leaflet precursors. This abnormality in the formation of the ICVS was associated with disruption of Notch-Jag1 signalling pathway, with Jag1^f/f^;Isl1-Cre mutants presenting with a similar phenotype.

**Conclusion:**

These data show that primary cilia, via the Notch-Jag1 signalling pathway, regulate differentiation of SHF cells in the aortic valve primordia. Additionally, we identify a mechanistic link between the developmental basis of quadricuspid and bicuspid arterial valve leaflets.

## Graphical Abstract

**Graphical Abstract:**
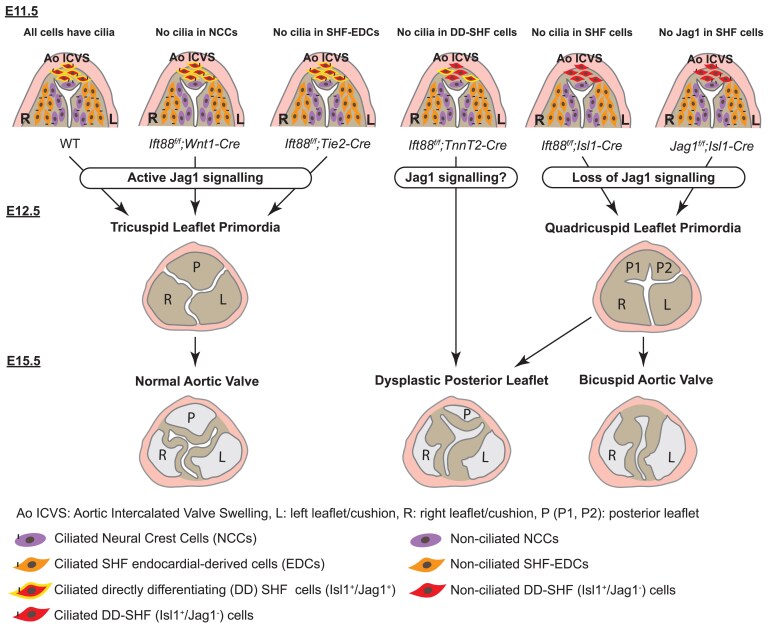

**Time of primary review: 19 days**

**See the editorial comment for this article ‘Loss of primary cilia triggers leaflet remodelling and bicuspid aortic valve formation’, by D. Marchese and S. Zaffran, https://doi.org/10.1093/cvr/cvaf150.**

## 1. Introduction

Structural abnormalities of the heart are the commonest human congenital malformations. There is good evidence for an underlying genetic aetiology, but the mechanisms underpinning the majority of these malformations remain undiscovered.^1^ It has been proposed that cilia, microtubular extensions found on almost all cells at least in culture, may be important in vertebrate development. Motile cilia are responsible for generating fluid flows across the ciliated embryonic node, which is fundamental to left–right specification.^2^ Disturbances of cilium function within the embryonic node are associated with reversal of organ positioning (situs inversus) and are likely to underlie abnormal left–right patterning in some patients with complex congenital heart disease (heterotaxy). It has also been suggested that subtle disruption of left–right patterning may account for some apparently non-syndromic congenital heart defects (CHD).^3^ Distinctly, the non-motile primary cilium, found on almost all cells, acts as a hub for receptor-ligand signalling and force transduction.^4,5^ It has also been suggested that disruption of cilium-related genes may be a significant cause of CHD.^6^

Whilst genomic studies have revealed that variants in cilial genes are enriched in rare familial cases, this is not the case for isolated *de novo* cases of CHD.^7,8^ Patients with known mutations or syndromes affecting cilia usually present with kidney or eye disease but relatively rarely have CHD.^9^ More recently, family-based genetic studies have linked mutations in cilial genes to valve malformations such as bicuspid aortic valve (BAV) and mitral valve prolapse.^10,11^ This has been supported by similar findings in mouse studies.^6,12,13^

*Ift88* (*intraflagellar transport 88*) is an intracellular transport protein required for ciliary biogenesis.^14^ In mice, complete knockout of *Ift88* is associated with a range of defects expected to occur following loss of cilia, including laterality disturbances that impact the heart; neural tube abnormalities; polydactyly; and other retinal, kidney, and brain defects.^15,16^ The hypomorphic Ift88 (*cobblestone*) mutant does not have situs inversus but exhibits a range of CHD including atrioventricular septal defect (AVSD), common arterial trunk, and hypoplasia of the ventricular myocardium.^17^ Tissue-specific knockout of Ift88 in the endocardium and its derivative cushion mesenchyme using Nfatc1-Cre resulted in BAV and thickened mitral valves^11,13^ although the mechanisms underpinning these malformations during early valvulogenesis were not described. Thus, the published data suggest that primary cilia may be required in specific cell types within the developing outflow tract and valves of the heart.

**Figure 1:**
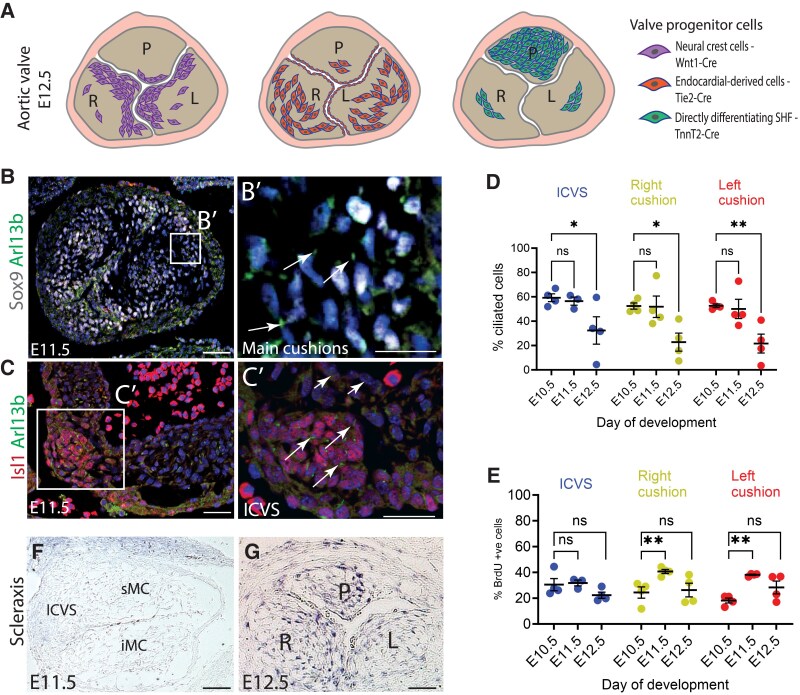
Dynamic presence of primary cilia in the main progenitor cells forming the arterial valves. (*A*) Schematic view of the different cell linage contributions to the three leaflet primordia of the aortic valve at E12.5.^18,19^ (*B-B’*) Representative immunofluorescent images showing primary cilia marked by Arl13b (green; arrows in *B’*) found on the cells of the main outflow cushions (labelled by Sox9; white) at E11.5 (*n* = 3). (*C-C’*) Representative immunofluorescent images of primary cilia labelled by Arl13b (green; arrows in *C’*) found on the cells of the ICVS (labelled by Isl1; red) at E11.5 (*n* = 3). In all cases, cell nuclei are labelled by DAPI (blue). (*D*) The percentage of ciliated cells found in the ICVS, and the left and right main cushions/leaflets followed the same dynamic pattern from E10.5 to E12.5, with the majority of cells bearing a cilium up to E11.5, with a drop to 20–30% at E12.5 (*n* = 4). Two-way ANOVA with Tukey test, mean ± SD. ns: not significant, *P < 0.05, **P < 0.01. (*E*) Quantification of proliferating cells labelled by BrdU showed no significant increase in proliferation at E12.5 that could account for the reduction in the percentage of ciliated cushion cells observed at E12.5 (*n* = 4). Two-way ANOVA with Tukey test, mean ± SD. ns: not significant, **P < 0.01. (*F*) Absence of expression of Scleraxis in the main cushions (sMC and iMC) and ICVS of the outflow tract at E11.5 (*n* = 3). (*G*) Expression of Scleraxis in the aortic valve leaflets at E12.5, suggesting up-regulation of VIC differentiation at this time point (*n* = 3). ICVS, intercalated valve swellings; sMC, superior main cushion; iMC, inferior main cushion; L, left leaflet; R, right leaflet; P, posterior leaflet. Scale bar = 100 μm.

Recent developmental studies have revealed many of the developmental processes that underlie valve formation. The leaflets found in the arterial valves have different embryological origins (*Figure 1A*). The left and right leaflets of both arterial valves (these are the coronary leaflets in the aortic valve) form from the distal part of the superior and inferior outflow tract cushions and are populated by invasion of neural crest cells (NCCs) and endothelial cells undergoing endothelial-to-mesenchymal transformation [referred to here as endothelial-derived cells (EDCs)]. These latter cells are originally derived from second heart field (SHF) progenitors. The posterior (non-coronary leaflet) of the aortic valve and the corresponding anterior leaflet of the pulmonary valve form directly from SHF progenitors via intermediary structures known as intercalated valve swellings (ICVS).^18,19^ We have previously reported that the ICVS differentiate directly from Isl1^+^ progenitor cells into Sox9-expressing valve interstitial cells (VICs) via a Notch-Jag-dependent mechanism.^18^ Thus, NCC, EDC (of SHF origin), and directly differentiating cells in the outflow wall (DD-SHF) all form VIC and are required for arterial valve morphogenesis. Our study, therefore, aimed to investigate the requirement for primary cilia in these different progenitor cells in the light of their various contributions to the formation of arterial valve leaflets.

In this study, we show that loss of *Ift88* in SHF cells expressing Isl1-Cre leads to formation of quadricuspid valve leaflet primordia, that later remodel into a bicuspid valve. The loss of cilia leads to disruption of Notch signalling, suggesting that this pathway may be cilia dependent for arterial valve development. Hence, this study uncovers a potential disease mechanism involved in the formation of BAV.

## 2. Methods

### 2.1 Mouse strains

*Ift88* flox (B6.129P2-Ift88tm1Bky/J;^20^) and *Jag1* flox (B6.129S-Jag1^tm2Grid^/SjJ;^21^) were obtained from Jackson Labs and genotyped as described previously. These were maintained on the C57Bl/6J background (Charles River Laboratories). *Wnt1-Cre*,^22^  *Tie2-Cre*,^23^  *Tnnt2-Cre*,^24^  *Mef2c-AHF-Cre*,^25^ and *Isl1-Cre*^26^ mice were inter-crossed with the *Ift88* flox mice to conditionally remove *Ift88* from the relevant lineage/cell type. *Jag1* flox mice were inter-crossed with *Isl1-Cre* mice to conditionally remove Jag1 from SHF cells. The reporter lines, *mTmG*^27^ or the *ROSA-Stop-eYFP*,^28^ were also crossed into these lines to follow the relevant lineage/cell type. Timed mattings were carried out overnight with the Cre allele introduced by male mice; the presence of a copulation plug was designated embryonic day (E) 0.5. Littermate controls were used where possible. Each experiment was carried out, at minimum, in triplicate. All animals were euthanized by CO_2_ inhalation followed by a secondary physical method, cervical dislocation. No anaesthesia was carried out on any animals in this study.

All mice were maintained according to standard laboratory conditions, and all procedures were carried out in accordance with the local Animal Welfare and Ethical Review Body, the UK Animals (Scientific Procedures) Act 1986, and Newcastle University (Project Licences 604533, P9E095FF4 and PP5418872) in line with Directive 2010/63/EU of the European Parliament. The Jag1;Isl1-Cre mouse study was approved by the local Animal Care and Use Committee of the First Faculty of Medicine, Charles University, in accordance with the local legislation and institutional requirements.

To calculate survival rates of different genotypes within litters, *Ift88^f/+^;Cre^+^* males were crossed with *Ift88^f/f^* females; thus, 25% *Ift88^f/f^;Cre^+^* offspring were expected. In the case of *Wnt1-Cre*, Ift88^f/+^;Wnt1-Cre^+^ males were crossed with either *Ift88^f/+^* or *Ift88^f/f^* females, generating *Ift88^f/f^;Wnt1-Cre^+^* mutant offspring at an expected ratio of 1:8 (12.5%) or 1:4 (25%), respectively. Mutant survival rates were calculated based on these ratios, with a *χ*^2^ test used to determine divergence from the expected ratios.

### 2.2 Histological analysis

Harvested tissues were rinsed in ice-cold phosphate-buffered saline (PBS) and fixed in 4% paraformaldehyde at 4°C, the duration of which depended on the age of the embryo, before paraffin embedding. Embryos were embedded in either a transverse, frontal, or sagittal orientation. For each analysis carried out using embryos, a minimum of three embryos for each developmental stage and section orientation were used (biological replicates). The analyses were carried out as separate experiments and a minimum of 5–10 sections (technical replicates) were analysed for consistency. For basic histological analysis, paraffin-embedded embryos were sectioned and stained with haematoxylin and eosin (H&E; Thermo Scientific, 6766010; Sigma, HHS-16) and alcian blue (Thermo Fisher Scientific; 40046-0025) following standard protocols. In some cases, slides were processed for H&E staining following immunofluorescence. In these cases, the slides were washed in water for 3 min before staining with haematoxylin for 10 min and then eosin for 5 min. This was followed by dehydration through a series of increasing concentrations of ethanol, then xylene, and finally mounting the slides with histomount (National Diagnostics, HS-103).

### 2.3 Immunofluorescence

Methodology has been published previously.^29^ Sections were cut from paraffin embedded embryos at 8 µm using a rotary microtome (Leica) in a transverse, frontal, or sagittal orientation. Slides were de-waxed with xylene (National Diagnostics) and rehydrated through a series of ethanol washes. Following washes in PBS, antigen retrieval was performed by boiling slides in citrate buffer (0.01 mol/L) pH 6.0 for 5 min. Sections were blocked in 10% fetal calf serum (FCS) at room temperature for 30 min and then incubated overnight at 4°C with the following primary antibodies diluted 1/100 in 2% FCS: acetylated-tubulin (Sigma-Aldrich; T5293), Ift88 (Merck, ABC932), Arl13b (Merck; SAB2700707), Isl1 (40.2D6, Developmental Studies Hybridoma Bank), GFP (abcam; ab13970, OriGene, TP401), eNOS (Santa Cruz; sc-654), Sox9 (abcam; ab185230 & Bio-Techne; AF3075), SM22 alpha (abcam; ab14106), cardiac Troponin I (HyTest; 4T21/2), Notch1ICD (Cell Signalling; 4147), Jagged1 (Cell Signalling; 2620), Notch2 (abcam; ab8296), Notch3 (abcam; ab23426), Lef1 (abcam; ab2230), Axin2 (abcam; ab32197), Caspase3 (abcam, 96615), Collagen I (abcam; ab21286), Versican (abcam; ab1032), Periostin (abcam; ab14041), and BrdU (abcam; ab6326). After washing, sections were incubated at room temperature for 1 h, with secondary antibodies (diluted 1/200 in 2% FCS) conjugated to either Alexa 488, Alexa 594, or Alexa 647 (Life Technologies). Fluorescent slides were then mounted with Vectashield Mounting medium with DAPI (Vector Labs). For fluorescent Notch1ICD staining, endogenous peroxidases were inhibited by treating slides with 3% H_2_O_2_ for 5 min, and then the slides were treated as if for immunofluorescence. Immunoreactivity of fluorescent Notch1ICD was observed using TSA™ Cyanine 3 System (Perkin Elmer NEL704A001KT). TBST was used for all washes and dilution of antibodies instead of PBS. Each experiment was carried out in triplicate using separate embryos (>3) and imaging multiple sections of the structure of interest where possible. Immunofluorescence images were collected using a Zeiss AxioImager Z1 fluorescence microscope equipped with Zeiss Apotome 2 (Zeiss, Germany). Acquired images were processed with AxioVision Rel 4.9 software.

### 2.4 Proliferation assay

Pregnant female mice were injected with BrdU (bromodeoxyuridine/5-bromo-2'-deoxyuridine; 19–160, Merck), intraperitoneally, at a concentration of 100 µg/g and were sacrificed after 2 h of treatment. The embryos were collected and fixed in 4% paraformaldehyde at 4°C and then processed for immunofluorescence.

### 2.5 Slide *in situ* hybridization

Methodology was carried out as previously described.^18^ Digoxigenin-labelled RNA antisense probes corresponding to the protein coding regions of Scleraxis (ENSG00000260428) were synthesized by T7 RNA polymerase (MAXIscript, Ambion) and *in vitro* transcription (pGEM_T easy, Promega), respectively. The slide *in situ* hybridization was performed using 10 mm paraffin-embedded sections of E11.5 and E12.5 mouse embryos. Slides were de-waxed with xylene and rehydrated with ethanol series washes. Slides were then fixed in 4% PFA for 20 min at room temperature prior to the treatment with proteinase K (10 mg/mL) for 10 min at 37˚C. Post-fixation by 4% PFA was performed at room temperature for 5 min. Slides were then processed to the acetylation reaction for 10 min in solution containing 100 mM triethanolamine, 40 mM hydrochloric acid, and 250 mM acetic anhydride. After that, pre-hybridization was performed for 2 h at 68˚C using hybridization buffer [50% formamide, 5× saline-sodium citrate buffer (SSC) (pH6), 200 mg/mL T-RNA, 100 mg/mL heparin, 0.1% Tween-20]; then, hybridization with RNA probes was carried out for 16 h. Following a series of washes, slides were blocked in 2% maleic acid buffer containing Tween 20 blocking solution (Roche) for 2 h and incubated with anti-digoxigenin-AP, fab fragments (Roche) overnight at 4˚C. Slides were then developed in NBT/BCIP solution for 18–36 h at room temperature. The reaction terminated through incubation in 100% methanol for 5 min, followed by dehydration with washes in a serial ethanol dilution (20–30 s per wash), incubation in xylene twice for 5 min, and finally mounting slides with histomount (Agar scientific). A minimum of three embryos at each stage and genotype were analysed.

### 2.6 RNAscope

RNAscope was performed using the RNAscope Multiplex Fluorescent Reagent Kit v2 from Bio-Techne (cat# 323100) according to the manufacturer’s instructions. Briefly, embryos were fixed in 4% PFA at 4°C overnight, serially dehydrated using ethanol, and embedded in paraffin oriented for transverse/frontal/sagittal sections. The embryos were then sectioned at 8 µm using a Leica microtome, and sections were mounted on positively charged slides (Marienfeld). The slides were then heated to 60°C for 10 min, allowed to cool to room temperature, and de-waxed in xylene for 6 min before dipping in 100% ethanol for 4 min and then air drying. After that, slides were incubated with hydrogen peroxide for 10 min at room temperature and rinsed in water before soaking in boiling target retrieval solution for 20 min using a steamer. After rinsing the slides in 100% ethanol and allowing to air dry, protease plus solution was added to the sections and incubated for 30 min at 40°C. RNA probes of Channel 2 (Ptch1, 402811-C2; Hey2, 404651-C2) were diluted in a 1 in 50 volume of Channel 1 probe (Gli3, 445511; Hes1, 417701) and incubated at 40°C for 2 h. All probes were from Bio-Techne. Following several sequential amplification and blocking steps, the slides were incubated in TSA Vivid Fluorophore Kit (7527/1 KIT, 7523/1 KIT, Bio-Techne) at a dilution of 1 in 1000 using TSA diluent. Finally, slides were mounted using ProLong gold antifade mounting (Thermo Fisher Scientific) and stored at −20°C until imaged.

### 2.7 Reconstruction from histological/immunofluorescent sections

Three-dimensional (3D) reconstructions of either H&E or immunofluorescence images were performed to elucidate and generate volumetric measurements of aortic valve leaflets at embryonic stages E11.5, E12.5, E13.5, and E15.5 using Amira Software (Thermo Fisher Scientific). Briefly, 8 μm sections were used to generate images for 3D reconstructions. A minimum of 10 images throughout the entirety of the aortic valve was imported to Amira Software for alignment and construction. Manual tracing of each individual leaflets using the Amira brush tool was followed by combining all traces to generate a 3D structure via the Amira generate surface tool.^30^ A total of 16 mutants and 16 littermate controls were analysed for BAV from different embryonic stages. Quantification of leaflets at E12.5 was performed using the default measurements in Amira. Data were then copied for statistical analysis and creation of graphs in GraphPad Prism 10. Where leaflet volumes were calculated, the position of the commissure, even if displaced towards the lumen, was used as the boundary between primordia and where two leaflets were present in the position of the posterior leaflet, they were counted together.

### 2.8 Quantification of the percentage of ciliated cells

Quantification of the percentage of cells carrying a cilium was performed on deconvoluted images (from Z-stack images) of the outflow tract at E10.5–E15.5 using the cell counter plugin in Fiji. Four representative images from three different embryos per developmental time point were used. Data were presented as the percentage of total cells bearing a cilium (percentage ciliated cells). Student’s two-tailed unpaired *t*-test was performed using GraphPad Prism 10.

### 2.9 Quantification of transcript/protein expression

The expression of Gli3, Ptch1, Lef1, N1ICD, Jag1, Hes1, and Hey2 was quantified by counting the cells that showed the expression of these markers as dots within their cell nuclei/membrane, in a representative 250 × 250 mm region of the ICVS of the aortic valve. The total nucleus number (marked with DAPI) within that region was also counted to represent the percentages of marker positive nuclei as a proportion of the total. Five sections from four controls and four *Ift88^f/f^;Isl1-Cre* mutant embryos at E10.5 and E11.5 were used to generate the data. Student’s (two-tailed unpaired) *t*-test was performed using GraphPad Prism 10 to compare between values.

### 2.10 Cell counts and statistical analysis

The nuclei of cells were manually counted in the aortic valve leaflet primordia at E11.5–E15.5, stained with H&E in which haematoxylin labelled cell nucleus for total cell counts, or immunofluorescent in which DAPI used for total cell counts, or Isl1 and Sox9 for cushion mesenchymal cell counts, from each of the three discrete primordia. In each case, a minimum of six representative sections from six different embryos were counted per time point. All sections were 8 μm in thickness and covered distal, mid, and proximal regions of aortic valve primordia. The data were presented as total cell counts for each leaflet or for all three leaflets together. Student’s (two-tailed unpaired) *t*-test, a one-way analysis of variance (ANOVA), or two-way ANOVA test were performed using GraphPad Prism 10.

## 3. Results

### 3.1 Primary cilia are present on the main progenitor cells forming the arterial valves

The presence of primary cilia on cells within the aortic valve primordia was confirmed using immunofluorescence with an Arl13b antibody at E10.5–E12.5 (*Figure 1B–D*). The majority of mesenchymal cells within the main cushions of the outflow tract possessed cilia at E10.5 and E11.5, as did Isl1-expressing cells in the ICVS (*Figure 1B* and *C*), but there was an approximately 50% reduction in the number of cells exhibiting cilia at E12.5, with a similar dynamic observed between the different primordia (*Figure 1D*). Cilia have been described in low shear stress areas on the endocardium of the chicken heart^31^; however, similar to Toomer *et al*.,^13^ we did not observe them in our study, possibly because the valve precursors are exposed to high levels of laminar shear stress. As there are both NCC and EDC in the main cushions, we used Wnt1-Cre and Tie2-Cre labelling to distinguish them and showed there were no differences in the percentage of ciliated cells between the two progenitors (see Supplementary material online, *Figure S1A*–*C*). The deciliation event at E12.5 was not correlated with an increase in proliferation, as measured by incorporation of BrdU, indicating that it was not linked to cells dissembling their cilium as they entered mitosis (*Figure 1E*). However, it did correlate with up-regulation of the expression of *Scleraxis* in the cushions at E12.5 (*Figure 1F* and *G*), suggesting it may be linked to differentiation of progenitors to VIC.^32^ Supporting this explanation, similar correlations between deciliation and differentiation were seen for myocardium and smooth muscle cell differentiation in the developing outflow tract (see Supplementary material online, *Figure S1D* and *E*).

### 3.2 Deletion of Ift88 in EDC and DD-SHF, but not NCC, results in cardiac abnormalities

**Figure 2:**
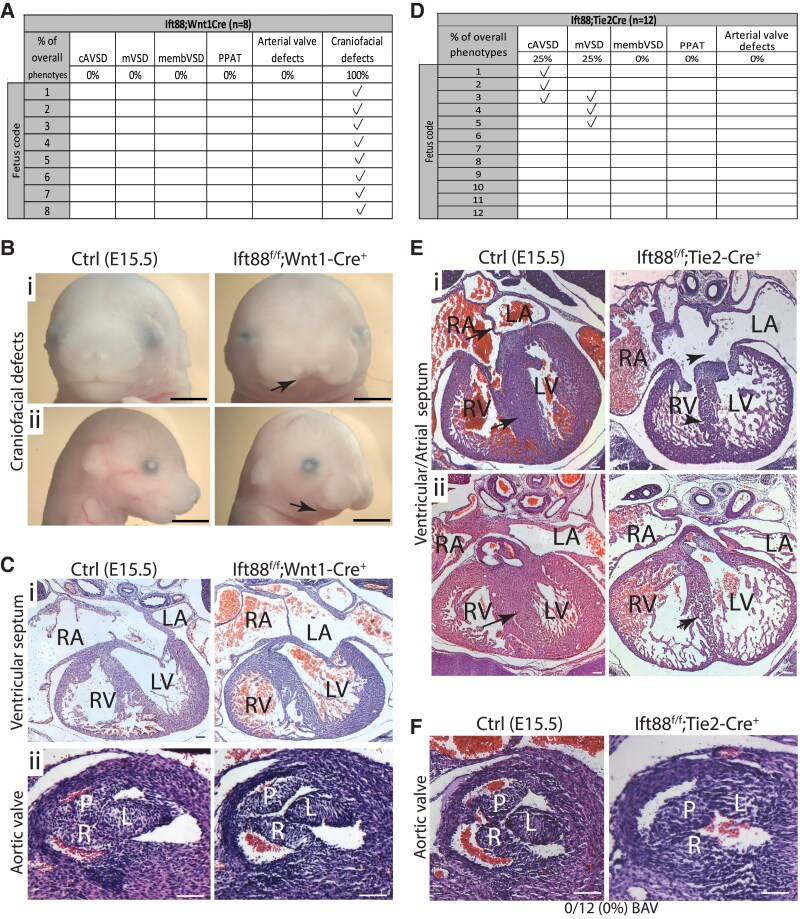
Primary cilia are not required in NCC- or EDC-derived cells for aortic valve development. (*A*) Table showing the different phenotypes observed within Ift88^ff^;Wnt1-Cre mutant foetuses at E15.5. (*B*) Craniofacial defects including frontonasal dysplasia (arrow in i) and hypoplastic mandible (arrow in ii) were observed in Ift88^f/f^;Wnt1-Cre mutants (*n* = 8/8) at E15.5. (*C*) Histological analysis of the hearts of E15.5 Ift88^f/f^;Wnt1-Cre foetuses showed no observable differences in ventricular septation (i) or in the aortic valve (ii) between Ift88^f/f^;Wnt1-Cre mutants and control littermates. *n* = 8 for each genotype. (*D*) Table showing cardiac malformations observed in Ift88^ff^;Tie2-Cre mutant foetuses at E15.5. (*E*) Histological analysis of Ift88^f/f^;Tie2-Cre mutants (*n* = 12) showing complete AVSD (arrowheads in i, *n* = 3/12) and muscular VSD (arrowhead in ii, *n* = 3/12). Arrows show the normal structures in control littermates. (*F*) The Ift88^ff^;Tie2-Cre aortic valve was comparable with control littermates in all foetuses examined (*n* = 12 for each genotype). cAVSD, complete atrioventricular septal defect; mVSD, muscular ventricular septal defects; membVSD, membranous ventricular septal defect; PPAT, parallel proximal arterial trunk; P, posterior leaflet; R, right leaflet; L, left leaflet; LV, left ventricle; RV, right ventricle; LA, left atrium; RA, right atrium. Scale bar = 100 μm (*C*, *E*, *F*) or 1 mm (*B*).

We next investigated the requirement for primary cilia in the developing outflow tract by conditional deletion of Ift88, as it is required for ciliogenesis and is present in primary cilia of outflow cushion cells (see Supplementary material online, *Figure S1F*). In Ift88^f/f^;Wnt1-Cre (conditional knockout in NCC), all foetuses examined (8/8) had craniofacial defects as previously described^33^ (*Figure 2A* and *B*). However, despite a reduction in the number of ciliated NCC within the pharyngeal arches of Ift88^f/f^;Wnt1-Cre mutants at E11.5 (see Supplementary material online, *Figure S2A*), no cardiovascular abnormalities were observed (*Figure 2C*). As this was a surprising finding, we checked whether NCCs in the outflow tract cushions normally express Ift88, confirming that they do (see Supplementary material online, *Figure S2C*). Thus, the absence of expression of Ift88 by cardiac NCC was not an explanation for the lack of a phenotype. Furthermore, the numbers of Ift88^f^;Wnt1-Cre embryos collected at E15.5 did not suggest there was intrauterine loss (see Supplementary material online, *Figure S2B*). Thus, the loss of Ift88 in NCC disrupted cilium formation and craniofacial development but had no effect on outflow tract development.

We next investigated whether loss of Ift88 in the endocardial lineage, using Tie2-Cre, would disrupt cardiac, and particularly arterial valve, morphogenesis. In contrast to the Wnt1-Cre experiments, 42% of Ift88^f/f^;Tie2-Cre mutants (5/12) exhibited cardiac defects (*Figure 2D* and *E*). Three mutants had complete AVSDs. Moreover, muscular ventricular septal defects, in the context of a thinned ventricular myocardium, were seen in 3/12 mutants (one of which also had AVSD). At E11.5, the atrioventricular cushions were underdeveloped and remained unfused in 2/6 cases, although the dorsal mesenchymal protrusion and primary atrial septum appeared well formed and fused with the superior atrioventricular cushion in all cases (see Supplementary material online, *Figure S2D*). Notably, there were no obvious abnormalities of the arterial valves in any of the twelve Ift88^f/f^;Tie2-Cre foetuses examined at E15.5, and the outflow cushions in mutants were comparable with controls at E11.5 (*Figure 2F*; Supplementary material online, *Figure S2D*). There was no evidence of that Ift88^f/f^;Tie2-Cre mutants were dying before E15.5 (see Supplementary material online, *Figure S2B*).

In the Ift88^f/f^;Tnnt2-Cre conditional knockout, which targets DD-SHF cells found in the ICVS, eight Ift88^f/f^;Tnnt2-Cre mutant foetuses were analysed for cardiac defects (see Supplementary material online, *Figure S3A*). Two had muscular VSDs, with one also having a membranous VSD, but there were no AVSDs. An additional foetus had parallel arterial trunks but no VSD (see Supplementary material online, *Figure S3B*). The aortic valve leaflets appeared thickened in 2/8 of Ift88^f/f^;Tnnt-Cre mutants (see Supplementary material online, *Figure S3C*), and in one of these cases, the right leaflet of the aortic valve had a cleft distally (confirmed by 3D reconstruction; Supplementary material online, *Figure S3C*). The pulmonary valve in one of the Ift88^f/f^;Tnnt2-Cre mutants also had thickening of all three leaflets; the remaining 7/8 mutants had a comparable pulmonary valve to their control littermates (see Supplementary material online, *Figure S3D*).

Together, these data show that there were no arterial valve malformations when Ift88 was deleted in NCC or in the EDC, but there were dysplastic arterial valve leaflets in some cases when Ift88 was deleted in the Tnnt2-Cre lineage. This suggests that cilia may be required for the development of the arterial valve leaflets, but not primarily in the NCC or EDC lineages.

### 3.3 Deletion of Ift88 in the Isl1Cre-targetted SHF leads to BAV

We next wanted to establish if earlier deletion of Ift88 (and thus cilia), in the undifferentiated SHF, would result in arterial valve defects. We first crossed Mef2c-AHF-Cre mice with the Ift88^f^ line. No arterial valve abnormalities were observed, although membranous VSD were seen in 2/8 mutant foetuses (see Supplementary material online, *Figure S4A*), as previously described in Burns *et al*.^12^ There was no evidence of intrauterine loss of Ift88^f/f^;Mef2c-AHF-Cre mutants (see Supplementary material online, *Figure S4B*).

**Figure 3:**
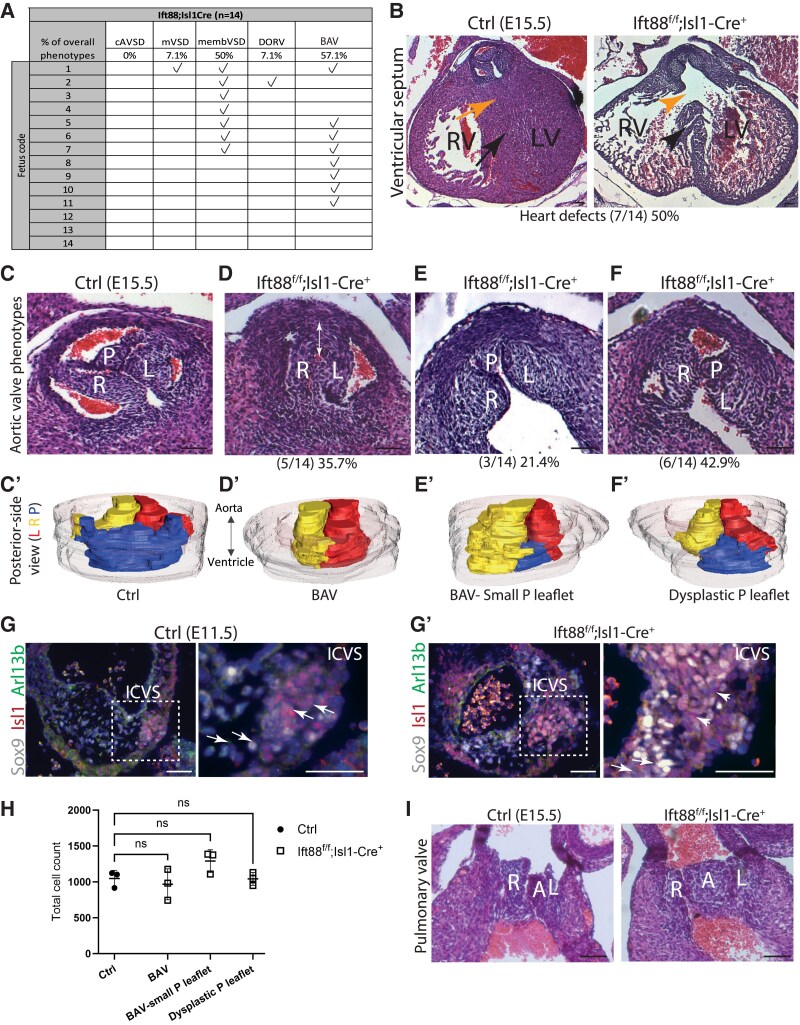
Loss of primary cilia in the SHF leads to abnormal aortic valve development. (*A*) Table showing cardiac malformations observed in Ift88^ff^;Isl1-Cre mutant foetuses at E15.5. (*B*) Histological analysis of Ift88^f/f^;Isl1-Cre at E15.5 (*n* = 14) revealed several congenital heart defects including membranous VSD (7/14; yellow arrow and arrowhead) and muscular VSD (1/14; black arrow). (*C–F*) BAV was observed in 8/14 of the Ift88^f/f^;Isl1-Cre foetuses at E15.5. Closer analysis of Ift88^f/f^;Isl1-Cre^+^ mutants, using histological sections and 3D reconstruction, revealed a spectrum of aortic valve malformations mainly affecting the posterior leaflet. In 5/8 mutants with BAV, the posterior leaflet was completely absent, with a large commissure found in the position of the missing leaflet (*D*, *D’*; white double headed arrow). In 3/8 of these mutants, the posterior leaflet only appeared in the proximal region of the valve, as a small leaflet with no associated sinus (*E*, *E’*). In the remaining 6/14 mutants with three leaflets, the posterior leaflet appeared dysplastic and the right leaflet relatively thickened. 3D reconstructions of the aortic valve showed that the posterior leaflet was shorter than the other two leaflets (compare *F’* with *C’*). (*G*, *G’*) Arl13b-expressing cilia are found on Isl1-expressing and Sox9-expressing cells in the ICVS in control embryos (*G*), whereas there are few if any cilia on the more abundant Sox9-expressing cells in the Ift88^f/f^;Isl1-Cre at comparable stages (*G’*). (*H*) Quantitation of total cell counts in the aortic valve leaflets across the various abnormalities identified no significant changes in the overall cell number at E15.5, *n* = 3 per group. One-way ANOVA with Tukey test, mean ± SD. ns: not significant. (*I*) Histological analysis of the pulmonary valve in Ift88^f/f^;Isl1-Cre^+^ mutants showed dysplastic anterior leaflet in 3/14 mutants. cAVSD, complete atrioventricular septal defect; mVSD, muscular ventricular septal defects; membVSD, membranous ventricular septal defect; DORV, double outlet right ventricle; L, left leaflet; P, posterior leaflet; R, right leaflet; A, anterior leaflet; LV, left ventricle; RV, right ventricle. Scale bar = 100 μm.

To examine an earlier requirement for Ift88 in the SHF, we turned to the Isl1-Cre line as this SHF lineage driver has a broader expression domain and is expressed earlier than Mef2c-AHF-Cre.^34^ Crosses between Ift88^f^ and Isl1Cre were carried out as for the other Cre lines. Cardiac abnormalities were present in 11 of 14 Ift88^f/f^;Isl1-Cre foetuses examined histologically at E15.5 (*Figure 3A*). Of these 11 mutants, one had double outlet right ventricle, one had muscular VSD, and seven had membranous VSD (*Figure 3A* and *B*); there were no cases of AVSD. Distinct from all other conditional knockouts of *Ift88*, BAV was apparent in 8/14 Ift88^f/f^;Isl1Cre mutant foetuses examined (*Figure 3D*, compare with 3C). Closer examination (that included 3D reconstruction) revealed that in all 14 Ift88^f/f^;Isl1-Cre mutants, there was a degree of disruption to the aortic valve leaflets, primarily affecting the posterior DD-SHF-derived leaflet. In five cases, the posterior leaflet was completely absent with a large commissure in the position where the missing leaflet would normally be located (*Figure 3D*). In three cases, there was an abnormally small posterior leaflet found only in the proximal part of the valve, with no associated sinus (*Figure 3E*). In the remaining six, whilst these were tri-leaflet aortic valves, the right coronary leaflet was thickened, and the posterior leaflet appeared to be mal-positioned and shorter (not extend the full length of arterial root) compared with controls (*Figure 3F*). Notably, Arl13b-expressing cilia were completely lost from Isl1^+^ SHF cells in the developing ICVS, although many cells (presumably NCC) still carried cilia in the main cushions (*Figure 3G*). Quantification of the total cell counts revealed that although there were various degrees of aortic valve malformation, the total number of cells was comparable between controls and mutants (*Figure 3H*), suggesting malformation might not be caused by changes to overall cell numbers but rather in the allocation of cells within the leaflets. Moreover, there were no reproducible differences in the expression of extracellular matrix molecules such as Collagen I, Versican, or Periostin at E18.5 or later at P1 (see Supplementary material online, *Figure S5A*), as has been reported for Ift88;Nfatc1-Cre mutants.^13^

No cases of bicuspid pulmonary valve were observed, but dysplastic pulmonary anterior leaflets were present in 3/14 cases examined (*Figure 3I*). As for the other lines, there was no evidence of death prior to E15.5 (see Supplementary material online, *Figure S4B*).

To explore why the deletion of Ift88 using Isl1-Cre produced arterial valve abnormalities whilst Mef2c-AHF-Cre did not, we examined the expression patterns of two Cre drivers within the developing outflow tract and arterial valves. Between E10.5 and E11.5, when the ICVS were first apparent, Isl1-Cre labelled all the cells in the ICVS as well as the myocardial and endocardial cells that overlay the ICVS (see Supplementary material online, *Figure S4C* and *D*). In contrast, whilst Mef2c-AHF-Cre labelled this myocardium and the endocardium, it did not label some cells within the ICVS (white and green arrowheads in Supplementary material online, *Figure S4C* and *D*). By E12.5, there was a subpopulation of cells in the endocardium and the underlying mesenchyme of the distal region of the posterior leaflet that was labelled by Isl1-Cre, but not Mef2c-AHF-Cre (see Supplementary material online, *Figure S4E*). Therefore, there are differences in the expression domains of Isl1-Cre and Mef2c-AHF-Cre that could potentially account for the differences observed when the different SHF-specific Cres are used to delete Ift88. Interestingly, the same region was labelled by both Cres in the pulmonary valve (see Supplementary material online, *Figure S4F*) suggesting that this cannot explain the milder defects observed in the pulmonary valve in Ift88^f/f^;Isl1-Cre foetuses. Therefore, although the differences in the expression patterns between Isl1-Cre and Mef2c-AHF-Cre could explain the differences in phenotypes, it could not account for the observed milder phenotypes in the pulmonary valves of Ift88^f/f^;Isl1-Cre.

### 3.4 Two leaflets are found in the position of the posterior leaflet in Ift88^f/f^;Isl1-Cre mutants

Having taken a systematic approach, knocking out Ift88 in NCC and EDC that together contribute to the mesenchyme of the main cushions, we showed that there was no aortic valve phenotype in either case and thus loss of cilia from the main cushions did not lead to BAV. Thus, although deletion of Ift88 using Isl1Cre would affect EDC^24^ and potentially some NCC,^35^ the valve anomalies were very unlikely to be due to the role of Ift88 in the main cushions. Thus, by a process of genetic subtraction, we homed in on the other Isl1-Cre-derived arterial valve precursors—the ICVS. We started by observing the morphological structure of the aortic ICVS at E11.5, as it is the precursor of the posterior leaflet that was mostly affected in Ift88^f/f^;Isl1-Cre mutants at E15.5.

**Figure 4:**
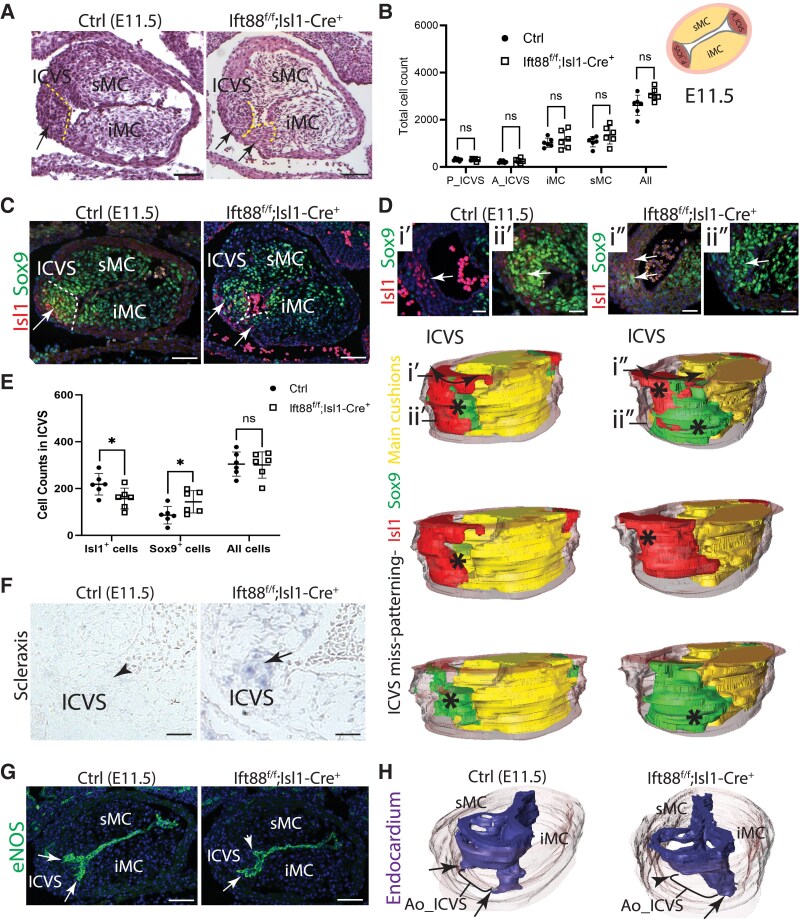
Premature differentiation of SHF cells correlates with abnormal ICVS formation in Ift88^f/f^;Isl1-Cre mutants during early development. (*A*) Histological analysis of the outflow tract in E11.5 Ift88^f/f^;Isl1-Cre mutants (*n* = 14), compared with their control littermates, revealed that although the ICVS was present in all cases, the mutant ICVS appeared to form two foci compared with the single focus in control littermates (12/14; arrows). (*B*) Quantification of total cell counts in the ICVS and main cushions (superior sMC and inferior iMC) at E11.5 showed comparable overall counts between Ift88^f/f^;Isl1-Cre mutants and their littermate controls, *n* = 6 for each genotype. Two-way ANOVA with Šídák test, mean ± SD. ns: not significant. (*C*) Matched immunofluorescence images for Isl1 (red; undifferentiated SHF cells) and Sox9 (green; partially differentiated cushion cells) confirmed the presence of two foci in the posterior ICVS (arrows in ii) of Ift88^f/f^;Isl1-Cre^+^ mutants. (*D*) 3D reconstructions of serial immunofluorescent images of the outflow tract, labelling main cushions (yellow) and ICVS with Isl1 (red) or Sox9 (green) revealed that the ICVS in Ift88^f/f^;Isl1-Cre^+^ mutants was broader (double headed arrows), with Sox9-expressing cells appearing to assemble a separate focus from the Isl1^+^ cells (asterisks). The expression of both Isl1 (red) and Sox9 (green) at the distal region (arrows in i’’) and the sole proximal expansion of Sox9^+^ cells (arrow in ii’’) in Ift88^f/f^;Isl1-Cre^+^ mutants; support the idea that there are two foci forming in the mutant ICVS. (*E*) Quantification of Sox9- and Isl1-expressing cells in the ICVS at E11.5 indicated significantly fewer Isl1^+^ cells, and more Sox9^+^ cells in the Ift88^f/f^;Isl1-Cre^+^ compared with that in control littermates, *n* = 6 for each genotype. Two-way ANOVA with Šídák test, mean ± SD. ns: not significant, *P < 0.05. (*F*) *In situ* hybridization showed that *Scleraxis* was highly expressed in the ICVS of 3/4 the Ift88^f/f^;Isl1-Cre^+^ mutants (arrow) at E11.5, whereas there was little or no expression of *Scleraxis* in any of the littermate controls at E11.5 (arrowhead, *n* = 4 for each genotype). (*G*, *H*) Labelling of the cushion/ICVS endocardium with eNOS (green) at E11.5 showed that the endocardium forms a regular arc over the aortic ICVS in the control heart (arrows), whereas it is foreshortened, reflecting the juxtaposition of the supernumerary ICVS with the adjacent cushion, in the Ift88^f/f^;Isl1-Cre^+^ mutant heart (arrowhead in *G*). This can also be seen in 3D reconstructions of the endocardium (*H*). ICVS, intercalated valve swellings; sMC, superior main cushion; iMC, inferior main cushion. Scale bar = 100 μm.

Histological sections of the E11.5 outflow tract identified that the posterior ICVS was present in all the mutants (*n* = 14/14), but in the majority of cases appeared to be split into two smaller structures (*n* = 12/14; *Figure 4A*). Cell counting for each of the primordia (cushions and ICVS) revealed no significant differences between controls and Ift88^f/f^;Isl1-Cre, suggesting that the defects were due to abnormal arrangement of cells, rather than an increase in cell number (*Figure 4B*). As at E11.5, the ICVS are composed of Isl1^+^ SHF progenitors that are differentiating to Sox9-expressing VIC^18^; we immunolabelled the cells in the ICVS with these markers. The immunostaining of the aortic ICVS with Isl1 (red) and Sox9 (green) supported the appearances of two foci in the mutants compared with the expected single focus in the control littermates. Moreover, whereas Sox9^+^ cells were found only centrally (at the vertex of the oval-shaped, frontally-cut outflow tract) in the wild-type embryos, they were found more laterally in the mutants resulting in the appearance of a broadened IVCS region (*Figure 4C*). Whilst the Isl1 and Sox9-expressing cells in the control ICVS were tightly overlapping to assemble a single cluster, the domain of ICVS in Ift88^f/f^;Isl1-Cre mutants was broader and, in some cases, formed two foci of cells (*Figure 4D*). Sox9-expressing cells accumulated in the proximal region of the outflow tract and assembled as a separate cluster from Isl1-expressing cells (*n* = 4; *Figure 4D*), supporting the idea that there were two foci in the mutant ICVS. To understand the relationship between these two populations of cells within the ICVS, quantification of these cells (Isl1^+^ and Sox9^+^ cells) was performed. This showed that there were fewer cells expressing Isl1 (red) alone and more cells expressing solely Sox9 (green), or co-expressing both Sox9 and Isl1, in Ift88^f/f^;Isl1-Cre mutants compared with controls (*n* = 6; *Figure 4E*). These data suggest premature differentiation of SHF progenitors to VIC in the ICVS of the Ift88^f/f^;Isl1-Cre mutants that led to fewer Isl1^+^ cells and more Sox9^+^ cells.

*In situ* hybridization for *Scleraxis*, which up-regulates with VIC differentiation, was observed in the ICVS of 3 of 4 Ift88^f/f^;Isl1-Cre mutants at E11.5, whereas this was not apparent in any of the control littermates (*Figure 4F*), supporting the idea that there is premature differentiation of DD-SHF cells to VIC in Ift88^f/f^;Isl1-Cre embryos. There was no evidence of premature differentiation to other derivatives of the SHF, such as myocardium or smooth muscle cells, in the outflow region of Ift88^f/f^;Isl1-Cre mutants (see Supplementary material online, *Figure S6A* and *B*). Altogether, this showed that the basis of the Ift88^f/f^;Isl1-Cre mutant phenotype at E11.5 was the formation of two foci within the ICVS, which most likely resulted from premature differentiation of SHF cells to VIC.

Immunolabelling of the endocardium with eNOS highlighted the abnormal shape and position of the forming ICVS in the Ift88^f/f^;Isl1-Cre embryos at E11.5, revealing that whereas the endocardium covering the ICVS formed a regular arc over the ICVS in wild-type embryos, this was more irregular in Ift88^f/f^;Isl1-Cre embryos, foreshortening towards the lumen in regions where there was a bulge of ICVS lateral to its normal position; thus the adjacent ICVS and cushion were in close apposition (*Figure 4G* and *H*). Examination of control embryos at this time point shows that the endocardium does not extend as far as the outflow wall and thus does not completely separate the ICVS and the main cushions (arrows in *Figure 4G* and *H*; supplementary material online, *Figure S6C*); this region of continuity was expanded in the Ift88^f/f^;Isl1-Cre embryos with no caspase-3-labelled cells observed in this region (see Supplementary material online, *Figure S6D*). Thus, the foreshortened endocardium in the Ift88^f/f^;Isl1-Cre embryos reflects the abnormal bulging of the ICVS in this position rather than any specific abnormality of the endocardium.

**Figure 5:**
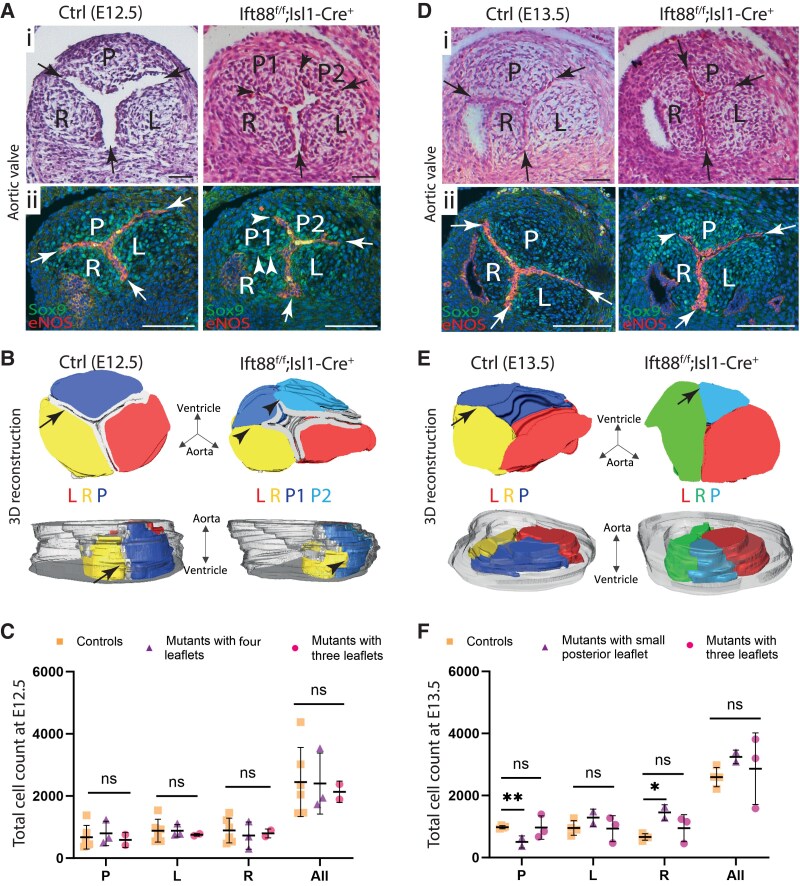
BAV in the Ift88^f/f^;Isl1-Cre mutants caused by remodelling of supernumerary leaflet primordia. (*A*) Histological analysis of the aortic valve leaflet primordia at E12.5 revealed the presence of four primordia in 3/6 Ift88^f/f^;Isl1-Cre mutants, compared with the usual three primordia seen in control littermates. (i) Arrows point to the commissures where adjacent leaflets attach to each other at the aortic wall, whereas arrowheads indicate displacement of commissures from the aortic wall towards the lumen. (ii) Immunolabelling of cushion mesenchyme (labelled by Sox9; green) and the endocardium overlying each leaflet primordium (labelled by eNOS; red) shows that all three commissures were close to the arterial wall in controls (arrows in ii) whereas at least one commissure was displaced towards the lumen in Ift88^f/f^;Isl1-Cre^+^ mutants (arrowheads in ii; *n* = 3/3. (*B*) 3D reconstructions, produced from anti-Sox9 and anti-eNOS immunofluorescence images, revealed the extent of the endocardium in each case (pale grey), relative to each of the primordia in the controls (P is blue, R is yellow, and L is red) and the Ift88^f/f^;Isl1-Cre^+^ mutants (two P are different shades of blue, R is yellow, and L is red). In the Ift88^ff^;Isl1-Cre mutants, the commissures were close to the lumen between the R and P1 primordium (arrowheads), with continuity in the mesenchyme between these conjoined primordia (arrowheads), *n* = 3/6. (*C*) Total cell counts from each of the aortic leaflet primordium revealed no significant differences between Ift88^f/f^;Isl1-Cre^+^ mutants and their littermate controls at E12.5. Where there was the appearance of two P primordia in the mutants, these were counted together (purple triangles) to allow comparison with controls. Mutants with three leaflet primordia were presented as pink circles. Two-way ANOVA with Šídák test, mean ± SD. ns: not significant. (*D*) Histological analysis of the aortic valve leaflets at E13.5 showed that the primordia were remodelling and could now be described as leaflets. A total of three leaflets, but with a small P leaflet, could be seen in two of seven mutants (arrows in i pointing at the commissures). Immunolabelling of mesenchymal cells with Sox9 (green) and the endocardium overlying each leaflet primordium (labelled by eNOS, red) again showed displacement of the commissure between P and the adjacent R leaflet (arrowhead in ii) towards the lumen. (*E*) 3D reconstructions, produced from anti-Sox9 immunofluorescence images without labelling of the endocardium, showed a small P leaflet (light blue) with a remodelling R leaflet (green) appearing to emerge more centrally (arrows) in Ift88^f/f^;Isl1-Cre mutants compared with that in controls (P is blue, R is yellow, and L is red), *n* = 2/7. (*F*) Total cell counts from each aortic leaflet revealed significant differences in the posterior and right leaflets between Ift88^f/f^;Isl1-Cre mutants and their littermate controls, but not in the overall cell counts for all three leaflets at E13.5. Mutants with small P leaflets are presented as purple triangles, whereas mutants with three leaflets are presented as pink circles. Two-way ANOVA with Šídák test, mean ± SD. ns: not significant, **P = 0.006, *P = 0.024. L, left leaflet; R, right leaflet; P, posterior leaflet; P1 and P2, super-numerary leaflets in the position of the usual posterior leaflet. Scale bar = 100 µm.

At E12.5, in the six examined mutants, 2/6 had aortic valves with three leaflets. 3D reconstructions of these cases identified that although there were three leaflets, the posterior leaflet appeared slightly smaller than that in the controls (see Supplementary material online, *Figure S7A*). However, comparison of the aortic valve leaflet volumes, based on data from the 3D reconstructions, between Ift88^f/f^;Isl1-Cre embryos and control littermates showed no significant differences between mutants and controls, thus no changes in the size of leaflet primordia at this stage (see Supplementary material online, *Figure S7B*). In 1/6 mutants, the leaflet primordia were developmentally delayed, as their appearances were similar to those of E11.5 hearts, with bulky leaflet primordia and unseptated outflow tract (see Supplementary material online, *Figure S7C*). In the remaining 3/6 cases examined at E12.5, four leaflets were seen, with large well-formed left and right main leaflet primordia but two small leaflets found in the posterior leaflet position (arrows and arrowheads *Figure 5A* and *B*). Furthermore, whilst the forming commissures in control aortic valves were positioned deep, close to the outflow tract wall, in the mutant quadricuspid valves they were shallow (*Figure 5A*; Supplementary material online, *Figure S7D*), reflecting the foreshortened endocardium that was observed where the supernumerary bulges of ICVS abutted the main cushions at E11.5 (compare with *Figure 4H*). Thus, there was no endocardium between forming leaflets in these regions at either E11.5 or E12.5. No caspase-3-labelled cells were observed in the forming leaflets of wild-type or mutant hearts at this or earlier time points (see Supplementary material online, *Figures S6D* and *S7E*), supporting the idea that there was no need for removal of endocardial cells from a remodelling fusion seam, as endocardial fusion between forming leaflets did not occur. 3D reconstructions of the aortic valve leaflets showed that the leaflets were grossly misshapen and confirmed the abnormal positioning of the commissures (*Figure 5B*). To investigate if hyperplasia was causing the supernumerary leaflets, quantification of cell numbers in the valve leaflet primordia (excluding the developmentally delayed mutant) revealed that there were no significant differences in the total number of cells within the forming valve leaflets between Ift88^f/f^;Isl1-Cre embryos and control littermates at E12.5 (*Figure 5C*). To clarify, if there were two posterior leaflet primordia in Ift88^f/f^;Isl1-Cre mutants, they were added together and presented as a separate group for these analyses so that they could be compared directly with controls. This suggested that whilst there were a subset of Ift88^f/f^;Isl1-Cre mutants with supernumerary leaflets in the position of the posterior leaflet, this was not due to increase in cell number within the leaflet primordia.

By E13.5, the arterial valve primordia were beginning to remodel in control hearts, with sinuses appearing between the leaflets and the vessel wall. As before, some Ift88^f/f^;Isl1-Cre mutants presented with three aortic leaflets (*n* = 3/7). However, whilst a representative 3D reconstruction of these mutant leaflets showed that the posterior leaflet appeared slightly smaller compared with that in controls (see Supplementary material online, *Figure S8A*), quantification of leaflet volume for these mutants revealed no significant differences compared with controls (see Supplementary material online, *Figure S8B*). In 2/7 mutants, and similar to the observed case at E12.5, the valve leaflets were developmentally delayed and appeared similar to those of E11.5 hearts with bulky cushion primordia and upseptated outflow tract (see Supplementary material online, *Figure S8C*). The remaining examined (2/7) mutants had large left and right leaflets, but only one small posterior leaflet (*Figure 5D*). 3D reconstructions of these valve leaflets (*Figure 5E*) confirmed the presence of a small posterior leaflet but showed an enlarged right leaflet that suggesting remodelling and fusion of the right main cushion primordia with one of the additional posterior primordia observed at E12.5. Interestingly, quantification of cells within the forming leaflets and their volume (for these two cases) suggested that although the total cell number and total leaflet volume for all leaflets together did not change, the posterior leaflet had fewer cells and reduced leaflet volume whilst the right leaflet had more cells and increased leaflet volume in the Ift88^f/f^;Isl1-Cre mutants compared with controls (*Figure 5F*; Supplementary material online, *Figure S8B*). This suggested that the changes in the architecture of the leaflets were not due to hypoplasia, but rather to altered cell distribution within the leaflets.

Together, these data suggest that a broadened and prematurely differentiating ICVS at E11.5 in Ift88^f/f^;Isl1-Cre mutants leads to the formation of two leaflet primordia in the position of the usual posterior leaflet at E12.5, pushed against the leaflet primordia from the adjacent main cushions. These ICVS-derived primordia, with some variation, merged with the adjacent right (and possibly left) leaflets between E13.5 and E15.5, resulting in BAV.

### 3.5 Hh and Wnt signalling are not altered in ICVS of Ift88^f/f^;Isl1-Cre mutants

To begin to uncover the mechanism of premature differentiation of the ICVS, we first investigated potential disruption to hedgehog (Hh) signalling as this is a recognized consequence of cilia loss.^36^ We therefore looked at the expression of Ptch1 and Gli3 in the developing ICVS at E11.5, when the aortic valve leaflet defects in the Ift88^f/f^;Isl1-Cre mutants first become apparent. RNAscope showed that both Ptch1 and Gli3 were expressed at similar levels in the ICVS at this time point (see Supplementary material online, *Figure S9A* and *B*; *n* = 3 for each genotype). Earlier analysis of the outflow region and immediately adjacent SHF in Ift88^f/f^;Isl1-Cre mutants and control littermates showed that although there was up-regulation of Ptch1 in the pharyngeal and paraxial mesoderm (*n* = 4 for each genotype), there were no changes in the distal OFT or the adjacent region of the SHF for Ptch1 or Gli3 (see Supplementary material online, *Figure S9C*).

Canonical Wnt signalling can also occur at the cilium^37^ so we examined the expression of Lef1, a readout of canonical Wnt signalling. Interestingly, whereas Lef1 was not expressed in the outflow tract at E10.5 (see Supplementary material online, *Figure S9D*), it specifically localized to the ICVS at E11.5 in both control and Ift88^f/f^;Isl1-Cre embryos (see Supplementary material online, *Figure S9E*). However, there were no differences in the numbers of Lef1-expressing cells between controls and mutants (*n* = 4; Supplementary material online, *Figure S9F*). We also looked at the expression of Axin2 as an additional readout of canonical Wnt signalling, but it did not localize to the developing ICVS (see Supplementary material online, *Figure S9G*). We therefore conclude there is no evidence that Hh or canonical Wnt signalling is impacted in the ICVS of Ift88^f/f^;Isl1-Cre embryos.

### 3.6 Notch signalling is disrupted in the ICVS of Ift88^f/f^;Isl1-Cre mutants

Notch signalling plays a major role in the development of the outflow tract.^38,39^ More specifically, loss of Notch signalling in the DD-SHF lineage results in loss of the posterior leaflet and BAV.^18^ Thus, we next examined the impact of loss of cilia on Notch signalling in the developing ICVS in Ift88^f/f^;Isl1-Cre mutants.

**Figure 6:**
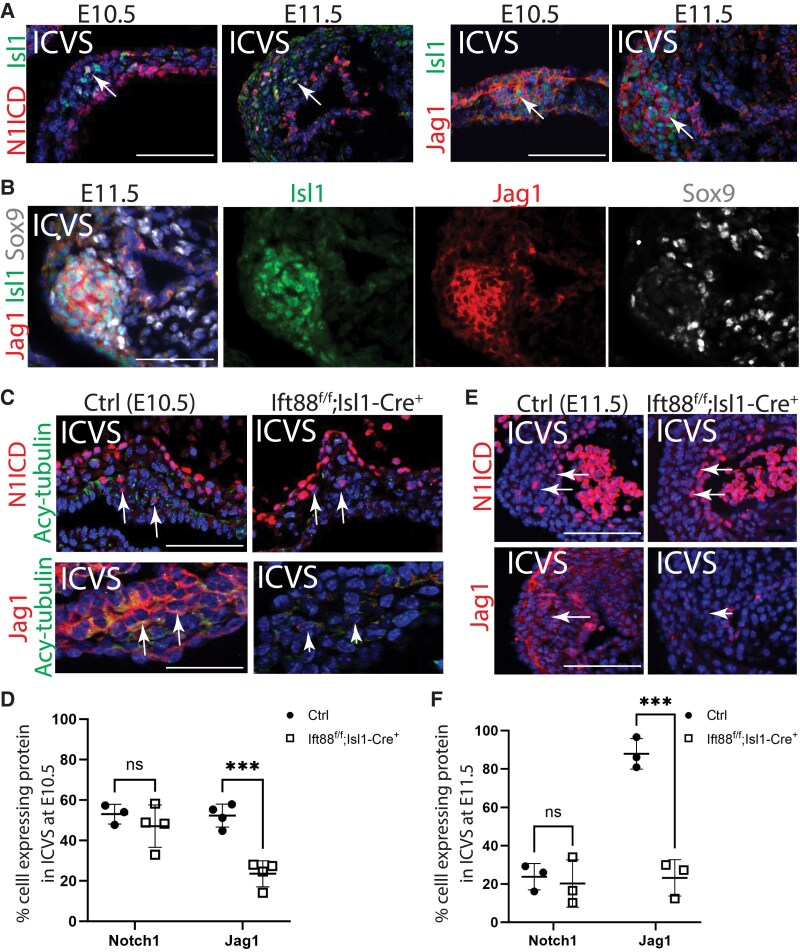
Disruption of Notch signalling pathway in the outflow tract of Ift88^f/f^;Isl1-Cre^+^ mutants. (*A*) Active Notch1 (N1ICD, red) was abundant in the ICVS at E10.5, co-localizing to Isl1-expressing cells (green), but was found in only a few ICVS cells by E11.5 (arrows). Jag1 (red) localized to Isl1-expressing cells (green) in the ICVS at both E10.5 and E11.5 (arrows). (*B*) The expression of Jag1 (red) is largely restricted to undifferentiated Isl1-expressing cells (green) in the ICVS at E11.5. (*C–E*) At E10.5 and E11.5, N1ICD in the ICVS was comparable between Ift88^f/f^;Isl1-Cre mutants and their littermate controls (arrows). In contrast, Jag1 showed reduced expression in the ICVS (arrowheads) compared with littermate controls (arrows) at both stages *n* = 3/3 (*C*, *E*). Quantification confirmed that whilst for N1ICD, there were no significant differences between mutants and control littermates in the number of positive cells expressing the receptor, the number of positive cells expressing Jag1 was dramatically reduced in the mutants compared with control littermates at E10.5 and E11.5 (*D*, *F*). Two-way ANOVA with Šídák test, mean ± SD. ns: not significant, ***P < 0.001. ICVS, intercalated valve swellings. Scale bar = 100 µm.

Using immunofluorescence, we examined the expression of active Notch1 (N1ICD), Notch2, Notch3, and Jag1 in the developing ICVS at E10.5 and E11.5. Whereas Notch1ICD and Jag1 were specifically localized to the developing ICVS both at E10.5 and E11.5 (*Figure 6A*), neither Notch2 nor Notch3 did at E10.5 or E11.5 (see Supplementary material online, *Figure S10A*). Previous studies have shown that both Notch1ICD and Jag1 localize to Isl1-expressing SHF cells in the developing ICVS^18^ (*Figure 6A* and *B*). As shown earlier (*Figure 6C*), these Isl1-expressing cells are ciliated.

Turning to the mutant line, we observed that whereas N1ICD expression appeared unaltered in the ICVS of Ift88^f/f^;Isl1-Cre embryos at E10.5, the number of cells expressing Jag1 was significantly reduced in the mutant ICVS at both E10.5 and E11.5 (*Figure 6C–F*), correlating with the reduced Isl1 expression and loss of cilia (see *Figure 3G*). This reduced Jag1 expression extended to the anterior and posterior region of the dorsal pericardial wall and distal walls of the outflow tract at E10.5 (see Supplementary material online, *Figure S10B*). Importantly, this abnormal expression occurred before the outflow tract appeared abnormal in the Ift88^f/f^;Isl1-Cre embryos and thus could be contributing to the later abnormalities. Hes1 and Hey2 are mediators of Notch signalling expressed in the developing outflow tract.^40^ Using RNAscope, we analysed the expression of Hes1 and Hey2 in the Ift88^f/f^;Isl1-Cre mutants compared with their littermate controls at E10.5. This revealed a marked reduction in the expression of Hes1 in the dorsal pericardial wall and distal outflow tract and Hey2 in the endothelial cells of the outflow tract in the mutants (*n* = 3/4; Supplementary material online, *Figure S10C* and *D*). Similar levels of Hes1 in the neural tube and Hey2 in the ventricle were found in Ift88^f/f^;Isl1-Cre mutants and controls (see Supplementary material online, *Figure S10E*), suggesting the reductions were specific to the Isl1-Cre-expressing regions of the embryo. Taken together, this suggests that a loss of Jag1-dependent signalling may lead to the premature differentiation of cells in the posterior ICVS that, following primordia remodelling, results in a smaller or absent posterior leaflet.

**Figure 7:**
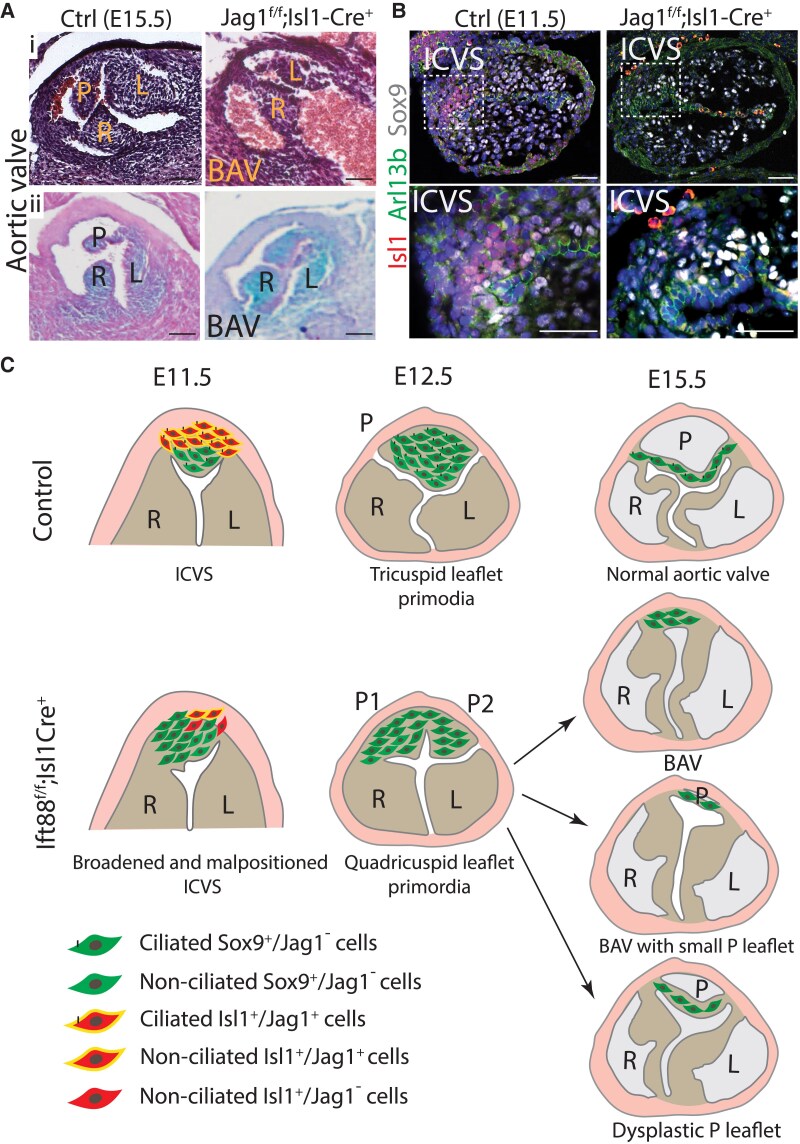
Aortic valve anomalies associated with premature differentiation of SHF cells in the ICVS of Jag1^f/f^;Isl1-Cre mutants. (*A*) Arterial valve defects, including BAV, in E15.5 Jag1^f/f^;Isl1-Cre mutants compared with littermate controls detected with H&E staining (i) and alcian blue (ii). (*B*) Immunofluorescence for Isl1 (red), Sox9 (white), and Arl13b (green) reveals that there are fewer Isl1^+^ cells, but more Sox9^+^ cells in the ICVS of the Jag1^f/f^;Isl1-Cre mutant compared with control littermates, *n* = 3. (*C*) Cartoon model illustrating how loss of primary cilia from Isl1-expressing cells disrupts aortic valve development and results in BAV. In controls, ICVS cells differentiate from Isl1-expressing progenitors (red) to VIC expressing Sox9 (green) as they lose Jag1 expression (yellow) between E11.5 and E12.5. They carry primary cilia during this period. Conversely, in mutants, when primary cilia are lost from the undifferentiated SHF cells (red), they lose their expression of Jag1 (yellow) and differentiate prematurely to VIC expressing Sox9 (green). This results in a broadened ICVS that merges with the main cushions leading to the appearance of BAV as the primordia remodel. L, left leaflet; R, right leaflet; P, posterior leaflet; ICVS, intercalated valve swellings. Scale bar = 100 µm.

To test the idea that Jag1 signalling in the SHF is required for normal development of the ICVS, we knocked it out in the Isl1 lineage. Examination of litters at E14.5–E16.5 revealed that 7 of 10 (70%) had abnormalities of the arterial valves, which included BAV (*Figure 7A*; Supplementary material online, *Figure S11A*). We then examined the developing ICVS at E11.5 in the Jag1^f/f^;Isl1-Cre mutants, to see if there was evidence for a broadened ICVS and/or premature differentiation of the SHF. Similar to the Ift88^f/f^;Isl1-Cre mutants, the ICVS of Jag1^f/f^;Isl1-Cre mutants had fewer Isl1^+^ cells and more dual-labelled Isl1^+^/Sox9^+^ and Sox9^+^ cells within the ICVS (*Figure 7B*; *n* = 3). Therefore, the observation of premature differentiation (fewer Isl1^+^ cells, more Sox9^+^ cells) in the Jag1^f/f^;Isl1-Cre mutants compared with controls suggests the involvement of Jag1 in regulating the differentiation of Isl1-positive cells within the ICVS to VIC during valve development. The implication is that Jag1, likely acting through cilia, is crucial for the proper timing and progression of this differentiation process.

## 4. Discussion

In this study, we have shown that primary cilia were found in the majority of cells in the early developing outflow tract but by E12.5, less than 50% were ciliated. Cells dissemble their primary cilium as they divide and reassemble them once cell division is complete.^36^ However, this ciliation event did not correlate with increases in proliferation of the forming valve leaflets. Instead, the decline in the percentage of ciliated mesenchymal cells in the forming valve leaflets correlated with the expression of *Scleraxis*, which is required for cushion cell differentiation.^32^ Cilium loss also correlated with differentiation of cardiomyocytes and smooth muscle cells in the developing outflow tract, suggesting that this process may be related to differentiation of at least some cell types. In support of a role for cilia in regulating cell differentiation, we observed premature differentiation of SHF-derived cells in the forming ICVS in Ift88^f/f^;Isl1-Cre mutants, ultimately leading to BAV, supporting the idea that cilia may be coordinating differentiation in the normal situation. It is known that some VICs are ciliated in the adult heart and that their loss, via deletion of Ift88, results in enlarged, myxomatous valves.^11^ Thus, it seems likely that cilia play roles in mature valves, potentially in maintaining valve homoeostasis.

In our hands, deletion of Ift88 from NCC or endocardial cells did not result in any outflow defects, despite effects on other parts of the embryo including other structures within the heart. With respect to NCC, our data are in agreement with previous authors, who reported severe craniofacial defects when Ift88 was deleted using Wnt1-Cre, but without mention of the outflow tract.^33^ However, our findings are at odds with the findings of Toomer *et al*.,^13^ who found a high incidence of BAV when Ift88 was knocked out in the endocardium using Nfatc1-Cre. As we observed reduced numbers of ciliated cells in the Tie2-Cre-positive cushion cells in the Ift88f;Tie2-Cre mutant foetuses, and other heart malformations were observed, it seems unlikely that the knockout was inefficient. It may be, however, that as we used different Cre drivers, differences in the penetrance or timing of Ift88 deletion explain the discrepancy. We did see arterial valve defects, however, when Ift88 was deleted using Tnnt2-Cre, which as well as targeting the myocardium, labels directly differentiating SHF (DD-SHF) that make a major contribution to the ICVS, with a more minor contribution to the right and left leaflets.^18^ Loss of cilia in this population gave rise to defects in the ventricular myocardium but also dysplastic arterial valve leaflets. Perhaps surprisingly, all three leaflets appeared to be affected equally, perhaps suggesting a role for cilia in the Tnnt2-Cre^+^ cells that are found in all three leaflets.^18^ Alternatively, there may be effects on the arterial valves secondary to gross abnormalities in the ventricular myocardium in the Ift88f;Tnnt2-Cre mutants.

When Ift88 was deleted using the SHF-specific driver Isl1-Cre, arterial valve defects were seen in more than half of the mutants. The observation that neither ourselves nor Burns *et al*.^12^ observed arterial valve defects following deletion of Ift88 with Mef2c-AHF-Cre, could be explained by a number of possibilities. Firstly, the Isl1-Cre allele^25^ that we use is a knock-in, meaning that mice carrying this allele are haplo-insufficient for Isl1. As it is Isl1-expressing cells in the ICVS that we believe are critical to the development of the phenotype in the Ift88^f/f^;Isl1-Cre mutants, reduced levels of Isl1 could have an impact. Arguing against this, we did not observe reduced expression of Isl1 in the Isl1-Cre^+^ hearts and the Isl1-Cre animals are not reported to have a phenotype.^25^ Alternatively, the observation of BAV in the Isl1-Cre, but not the Mef2c-AHF-Cre mutants, could reflect a difference in the precise cells labelled by each Cre driver, related to timing or precise pattern of expression. Close examination of the expression domains showed that at E11.5 and E12.5, there was a population of cells in the valve endocardium and underlying mesenchyme of the aortic ICVS that was labelled by Isl1-Cre, but not by Mef2c-AHF-Cre. Whether either of these differences is enough to explain the difference between the phenotypes when Ift88 is knocked out using the two Cre drivers, or whether it relates to earlier differences in pattern or timing of activation, remains to be shown. The observation that Isl1-Cre labels a sub-population of NCC in the outflow cushions^35^ seems unlikely to be the explanation, as loss of cilia from all cells using Wnt1-Cre as a driver did not result in outflow abnormalities.

There are several suggested mechanisms for how BAV may arise.^41,42^ Moreover, it has been suggested that there may be an ontological link between quadricuspid and bicuspid leaflets. Clinically, quadricuspid aortic valve (QAV) is found in up to 0.04% of the population,^43^ so is much less common than BAV. Interestingly, QAV and BAV are both reported in a strain of Syrian hamsters.^44^ Moreover, we have previously described the presence of two leaflet primordia in the position of the normal posterior leaflet in the ROCKDN;Wnt1-Cre mouse mutant, where both BAV and QAV were observed at E15.5.^29^ Here, in Ift88^f/f^;Isl1-Cre mutants, although we did not observe any cases with QAV at E15.5, a subset of Ift88^f/f^;Isl1-Cre had the appearance of quadricuspid leaflet primordia at early stages. At E11.5, the ICVS of these Ift88^f/f^;Isl1-Cre were broader with premature differentiation of Isl1^+^ progenitors to Sox9-expressing VIC that formed two separated foci within the region where the posterior leaflet forms. Presumably, these two foci in the ICVS were the precursors of the two small posterior leaflet primordia that were observed by E12.5. Close apposition of the ICVS and the main cushions, prior to the formation of endocardium between them, then results in the appearance of four forming leaflets at the lumenal surface of the forming valve, but continuity of mesenchyme between them more laterally. As development proceeds, at E12.5 and E13.5, there is thus no need to remodel an endocardial fusion seam, as such a fusion seam never existed, with the fusion occurring prior to the normal extension of the endocardium towards the walls of the arterial roots to cover the entirety of the forming valve leaflet. In the healthy developing aortic valve, the leaflet primordia are arranged in close proximity and occlude the outflow tract between heartbeats.^45^ It seems likely that the presence of an additional primordium leads to excessive apposition between adjacent primordia, resulting in merging of adjacent primordia as the leaflets continue to remodel, leading to BAV. It is unclear whether a broadened ICVS is a common model for the formation of quadricuspid leaflets, but the most common patterns of QAV in human have supernumerary leaflets in the position of the normal posterior leaflet,^46–48^ similar to what we observe. Thus, a mechanism where a broadened ICVS leads to the formation of two leaflets in the position of the usual posterior leaflet may be clinically relevant.

A number of different signalling pathways rely on cilia to transduce the signal from the cell surface to the nucleus, the best known of these is the Hh pathway.^49^ Sonic Hh (Shh) signalling is known to be important for outflow tract development^50^ and loss of cilia abrogates Shh signalling.^49^ However, there do not appear to be any direct associations in the published literature between Shh signalling and arterial valve development, although there is a link between Desert Hh signalling and development of the mitral valve.^10^ Surprisingly, despite the dramatic reduction in cilia numbers and length we observed in Ift88^f/f^;Isl1-Cre mutants compared with their control littermates, we did not find reproducible differences in the levels or distribution of the Shh signalling components Ptch1 and Gli3 in the ICVS or outflow tract, although Ptch1 was reproducibly up-regulated in the pharyngeal and paraxial mesoderm in the mutants. Canonical Wnt signalling has also been associated with cilia^36^ and has been linked to valve development.^51^ Despite this, and the specific expression of Lef1, a canonical Wnt marker, in the developing ICVS at E11.5, we did not find any differences in the numbers of cells expressing Lef1 in the Ift88^f/f^;Isl1-Cre embryos; other markers we tested were not expressed in the developing ICVS. Thus, although not exhaustive, we could find no evidence that canonical Wnt signalling was altered by the loss of cilia in our study.

In contrast to the data for Hh and Wnt signalling, we saw a significant reduction in the Notch ligand Jag1 in the ICVS at E11.5 and in the distal outflow tract and the adjacent pericardial wall at E10.5. Hes1 and Hey2, downstream mediators of Notch signalling, were also down-regulated in the outflow region at E10.5. Thus, we suggest a mechanism where loss of cilia in the cells of the forming ICVS leads to their premature differentiation and formation of two primordia in the position of the usual posterior leaflet. Although not as well described as Hh, there is significant evidence for a link between cilia and Notch signalling (reviewed in Wheway *et al*., Pala et al., and Yanardag and Pugacheva^36,52,53^), and loss of cilia has been associated with abrogation of Notch signalling in a number of scenarios.^54,55^ In the outflow tract, loss of cilia in Ift88^f/f^;Isl1-Cre embryos resulted in a reduction in the levels of Jag1, Hes1, and Hey2, all of which have been shown to be important for outflow tract development, and specifically in the case of Jag1, the arterial valves.^18,56–58^ Indeed, conditional deletion of Jag1/Jag2 in DD-SHF cells in the ICVS, using Tnnt2-Cre, resulted in BAV characterized by absence of the posterior leaflet, similar to what we describe for the Ift88^f/f^;Isl1-Cre mutants. To add to this, we now show that deletion of Jag1 in the SHF, using Isl1-Cre as a driver, results in very similar arterial valve defects as when cilia are removed from the SHF-derived cells in the Ift88^f/f^;Isl1-Cre embryos. Moreover, the mechanism appears to be similar, as in both cases, there was a broadened ICVS and premature differentiation of Isl1-expressing SHF cells to Sox9-expressing VIC at E11.5. This provides strong supporting evidence for the idea that Jag1 signalling is disrupted by loss of cilia in the SHF, and this impacts directly on the developing ICVS through premature differentiation, resulting in BAV (*Figure 7C*).

Large-scale ENU mouse screens for mutants with CHDs have suggested a high burden of cilia-related genes.^6^ However, whilst genomic studies have suggested that variants in cilial genes are enriched in rare familial cases, this is not the case for isolated *de novo* cases of CHD,^7,8^ raising the possibility that the interpretation of the mouse data was biased by the extensive delineation of the ciliome and the associated high number of gene ontology terms relating to cilia. Notably, almost 1000 genes have been associated with cilia,^59^ comparable with the 1200 genes reported to be expressed in mitochondria,^60^ another well-studied organelle. Another possibility is that disruption of cilial genes may allow intrauterine development in mouse but may be lethal early in development in human, and hence never be encountered in human medical practice. It is important to recognize that the malformations seen in the Ift88^ff^;Tie2-Cre and Ift88^ff^;Tnnt2-Cre foetuses included major disruptions to myocardial architecture that may well be embryonic lethal during human pregnancy. In contrast, the malformations found in Ift88^ff^;Isl1-Cre, principally, BAV, are compatible with survival *in utero*, and hence, the case for a role for cilia in valve malformations, especially the aortic valve, is well made. Thus, early developmental loss due to non-survivable cardiac malformations may be a factor influencing the paucity of heart malformations in ciliopathy syndromes.

**Translational perspective**

Several genomic studies in human and mouse have suggested that disruption of cilium-related genes may be a significant cause of congenital heart defects (CHD). Although there are limited data from animal models to suggest a link between cilia and bicuspid aortic valve (BAV), the mechanisms underpinning BAV formation during early valvulogenesis have not been described. Here, we established a potential mechanism underpinning BAV formation, highlighting a role for primary cilia in a subset of valve interstitial cells (VICs) derived from second heart field progenitors. Loss of cilia altered VIC differentiation and valvulogenesis. This study confirms that disruption of cilial formation and/or function can lead to arterial valve defects and could pave the way to finding therapies for patient benefit.

## Supplementary Material

cvaf108_Supplementary_Data

## Data Availability

All data that support the findings not presented in main figures and supplementary files are available from the corresponding author upon request.

## References

[1] Pierpont ME, Brueckner M, Chung WK, Garg V, Lacro RV, McGuire AL, Mital S, Priest JR, Pu WT, Roberts A, Ware SM, Gelb BD, Russell MW. Genetic basis for congenital heart disease: revisited: a scientific statement from the American Heart Association. Circulation 2018;138:e653–e711. 30571578 10.1161/CIR.0000000000000606 PMC6555769

[2] Shinohara K, Hamada H. Cilia in left–right symmetry breaking. Cold Spring Harb Perspect Biol 2017;9:a028282. 28213464 10.1101/cshperspect.a028282 PMC5629998

[3] Versacci P, Pugnaloni F, Digilio MC, Putotto C, Unolt M, Calcagni G, Baban A, Marino B. Some isolated cardiac malformations can be related to laterality defects. J Cardiovasc Dev Dis 2018;5:24. 29724030 10.3390/jcdd5020024 PMC6023464

[4] Mill P, Christensen ST, Pedersen LB. Primary cilia as dynamic and diverse signalling hubs in development and disease. Nat Rev Genet 2023;24:421–441. 37072495 10.1038/s41576-023-00587-9 PMC7615029

[5] Hilgendorf KI, Myers BR, Reiter JF. Emerging mechanistic understanding of cilia function in cellular signalling. Nat Rev Mol Cell Biol 2024;25:555–573. 38366037 10.1038/s41580-023-00698-5 PMC11199107

[6] Li Y, Klena NT, Gabriel GC, Liu X, Kim AJ, Lemke K, Chen Y, Chatterjee B, Devine W, Damerla RR, Chang C, Yagi H, San Agustin JT, Thahir M, Anderton S, Lawhead C, Vescovi A, Pratt H, Morgan J, Haynes L, Smith CL, Eppig JT, Reinholdt L, Francis R, Leatherbury L, Ganapathiraju MK, Tobita K, Pazour GJ, Lo CW. Global genetic analysis in mice unveils central role for cilia in congenital heart disease. Nature 2015;521:520–524. 25807483 10.1038/nature14269 PMC4617540

[7] Karp N, Grosse-Wortmann L, Bowdin S. Severe aortic stenosis, bicuspid aortic valve and atrial septal defect in a child with Joubert Syndrome and Related Disorders (JSRD)—a case report and review of congenital heart defects reported in the human ciliopathies. Eur J Med Genet 2012;55:605–610. 22910529 10.1016/j.ejmg.2012.07.010

[8] Watkins WS, Hernandez EJ, Wesolowski S, Bisgrove BW, Sunderland RT, Lin E, Lemmon G, Demarest BL, Miller TA, Bernstein D, Brueckner M, Chung WK, Gelb BD, Goldmuntz E, Newburger JW, Seidman CE, Shen Y, Yost HJ, Yandell M, Tristani-Firouzi M. *De novo* and recessive forms of congenital heart disease have distinct genetic and phenotypic landscapes. Nat Commun 2019;10:4722. 31624253 10.1038/s41467-019-12582-y PMC6797711

[9] Braun DA, Hildebrandt F. Ciliopathies. Cold Spring Harb Perspect Biol 2017;9:a028191. 27793968 10.1101/cshperspect.a028191 PMC5334254

[10] Fulmer D, Toomer K, Guo L, Moore K, Glover J, Moore R, Stairley R, Lobo G, Zuo X, Dang Y, Su Y, Fogelgren B, Gerard P, Chung D, Heydarpour M, Mukherjee R, Body SC, Norris RA, Lipschutz JH. Defects in the exocyst-cilia machinery cause bicuspid aortic valve disease and aortic stenosis. Circulation 2019;140:1331–1341. 31387361 10.1161/CIRCULATIONAHA.119.038376 PMC6989054

[11] Toomer KA, Yu M, Fulmer D, Guo L, Moore KS, Moore R, Drayton K'D, Glover J, Peterson N, Ramos-Ortiz S, Drohan A, Catching BJ, Stairley R, Wessels A, Lipschutz JH, Delling FN, Jeunemaitre X, Dina C, Collins RL, Brand H, Talkowski ME, Del Monte F, Mukherjee R, Awgulewitsch A, Body S, Hardiman G, Hazard ES, da Silveira WA, Wang B, Leyne M, Durst R, Markwald RR, Le Scouarnec S, Hagege A, Le Tourneau T, Kohl P, Rog-Zielinska EA, Ellinor PT, Levine RA, Milan DJ, Schott J-J, Bouatia-Naji N, Slaugenhaupt SA, Norris RA. Primary cilia defects causing mitral valve prolapse. Sci Transl Med 2019;11:eaax0290. 31118289 10.1126/scitranslmed.aax0290 PMC7331025

[12] Burns TA, Deepe RN, Bullard J, Phelps AL, Toomer KA, Hiriart E, Norris RA, Haycraft CJ, Wessels A. A novel mouse model for cilia-associated cardiovascular anomalies with a high penetrance of total anomalous pulmonary venous return. Anat Rec 2019;302:136–145. 10.1002/ar.23909 PMC6312498 30289203

[13] Toomer KA, Fulmer D, Guo L, Drohan A, Peterson N, Swanson P, Brooks B, Mukherjee R, Body S, Lipschutz JH, Wessels A, Norris RA. A role for primary cilia in aortic valve development and disease. Dev Dyn 2017;246:625–634. 28556366 10.1002/dvdy.24524 PMC5548133

[14] Pazour GJ, Baker SA, Deane JA, Cole DG, Dickert BL, Rosenbaum JL, Witman GB, Besharse JC. The intraflagellar transport protein, IFT88, is essential for vertebrate photoreceptor assembly and maintenance. J Cell Biol 2002;157:103. 11916979 10.1083/jcb.200107108 PMC2173265

[15] Clement CA, Kristensen SG, Møllgård K, Pazour GJ, Yoder BK, Larsen LA, Christensen ST. The primary cilium coordinates early cardiogenesis and hedgehog signaling in cardiomyocyte differentiation. J Cell Sci 2009;122:3070–3082. 19654211 10.1242/jcs.049676 PMC2729259

[16] Lehman JM, Michaud EJ, Schoeb TR, Aydin-Son Y, Miller M, Yoder BK. The Oak Ridge Polycystic Kidney mouse: modeling ciliopathies of mice and men. Dev Dyn 2008;237:1960–1971. 18366137 10.1002/dvdy.21515 PMC2677030

[17] Willaredt MA, Gorgas K, Gardner HAR, Tucker KL. Multiple essential roles for primary cilia in heart development. Cilia 2012;1:23. 23351706 10.1186/2046-2530-1-23 PMC3563622

[18] Eley L, Alqahtani AMS, Macgrogan D, Richardson RV, Murphy L, Salguero-Jimenez A, Sintes Rodriguez San Pedro M, Tiurma S, McCutcheon L, Gilmore A, de La Pompa JL, Chaudhry B, Henderson DJ. A novel source of arterial valve cells linked to bicuspid aortic valve without raphe in mice. eLife 2018:7:e34110. 29956664 10.7554/eLife.34110 PMC6025960

[19] Mifflin JJ, Dupuis LE, Alcala NE, Russell LG, Kern CB. Intercalated cushion cells within the cardiac outflow tract are derived from the myocardial troponin T Type 2 (Tnnt2) Cre lineage. Dev Dyn 2018;247:1005–1017. 29920846 10.1002/dvdy.24641 PMC6105448

[20] Haycraft CJ, Zhang Q, Song B, Jackson WS, Detloff PJ, Serra R, Yoder BK. Intraflagellar transport is essential for endochondral bone formation. Development 2007;134:307–316. 17166921 10.1242/dev.02732

[21] Kiernan AE, Xu J, Gridley T. The Notch ligand JAG1 is required for sensory progenitor development in the mammalian inner ear. PLoS Genet 2006;2:e4. 16410827 10.1371/journal.pgen.0020004 PMC1326221

[22] Danielian PS, Muccino D, Rowitch DH, Michael SK, McMahon AP. Modification of gene activity in mouse embryos *in utero* by a tamoxifen-inducible form of Cre recombinase. Curr Biol 1998;8:1323–1326. 9843687 10.1016/s0960-9822(07)00562-3

[23] Kisanuki YY, Hammer RE, Miyazaki J, Williams SC, Richardson JA, Yanagisawa M. Tie2-Cre transgenic mice: a new model for endothelial cell-lineage analysis in vivo. Dev Biol 2001;230:230–242. 11161575 10.1006/dbio.2000.0106

[24] Jiao K, Kulessa H, Tompkins K, Zhou Y, Batts L, Baldwin HS, Hogan BLM. An essential role of Bmp4 in the atrioventricular septation of the mouse heart. Genes Dev 2003;17:2362–2367. 12975322 10.1101/gad.1124803 PMC218073

[25] Verzi MP, McCulley DJ, De Val S, Dodou E, Black BL. The right ventricle, outflow tract, and ventricular septum comprise a restricted expression domain within the secondary/anterior heart field. Dev Biol 2005;287:134–145. 16188249 10.1016/j.ydbio.2005.08.041

[26] Yang L, Cai CL, Lin L, Qyang Y, Chung C, Monteiro RM, Mummery CL, Fishman GI, Cogen A, Evans S. Isl1Cre reveals a common Bmp pathway in heart and limb development. Development 2006;133:1575. 16556916 10.1242/dev.02322 PMC5576437

[27] Muzumdar MD, Tasic B, Miyamichi K, Li N, Luo L. A global double-fluorescent cre reporter mouse. Genesis (United States) 2007;45:593–605. 10.1002/dvg.20335 17868096

[28] Srinivas S, Watanabe T, Lin CS, William CM, Tanabe Y, Jessell TM, Costantini F. Cre reporter strains produced by targeted insertion of EYFP and ECFP into the ROSA26 locus. BMC Dev Biol 2001;1:1–8. 11299042 10.1186/1471-213X-1-4 PMC31338

[29] Phillips HM, Mahendran P, Singh E, Anderson RH, Chaudhry B, Henderson DJ. Neural crest cells are required for correct positioning of the developing outflow cushions and pattern the arterial valve leaflets. Cardiovasc Res 2013;99:452–460. 23723064 10.1093/cvr/cvt132 PMC3718324

[30] Garza-Lopez E, Vue Z, Katti P, Neikirk K, Biete M, Lam J, Beasley HK, Marshall AG, Rodman TA, Christensen TA, Salisbury JL, Vang L, Mungai M, AshShareef S, Murray SA, Shao J, Streeter J, Glancy B, Pereira RO, Abel ED, Hinton A Jr. Protocols for generating surfaces and measuring 3d organelle morphology using amira. Cells 2022;11:65. 10.3390/cells11010065 PMC8750564 35011629

[31] Van Der Heiden K, Groenendijk BCW, Hierck BP, Hogers B, Koerten HK, Mommaas AM, Gittenberger-de Groot AC, Poelmann RE. Monocilia on chicken embryonic endocardium in low shear stress areas. Dev Dyn 2006;235:19–28. 16145662 10.1002/dvdy.20557

[32] Levay AK, Peacock JD, Lu Y, Koch M, Hinton RB, Kadler KE, Lincoln J. Scleraxis is required for cell lineage differentiation and extracellular matrix remodeling during murine heart valve formation in vivo. Circ Res 2008;103:948–956. 18802027 10.1161/CIRCRESAHA.108.177238 PMC2743952

[33] Tian H, Feng J, Li J, Ho T-V, Yuan Y, Liu Y, Brindopke F, Figueiredo JC, Magee W, Sanchez-Lara PA, Chai Y. Intraflagellar transport 88 (IFT88) is crucial for craniofacial development in mice and is a candidate gene for human cleft lip and palate. Hum Mol Genet 2017;26:860–872. 28069795 10.1093/hmg/ddx002 PMC6075526

[34] Black BL . Transcriptional pathways in second heart field development. Semin Cell Dev Biol 2007;18:67–76. 17276708 10.1016/j.semcdb.2007.01.001 PMC1855211

[35] Engleka KA, Manderfield LJ, Brust RD, Li L, Cohen A, Dymecki SM, Epstein JA. Islet1 derivatives in the heart are of both neural crest and second heart field origin. Circ Res 2012;110:922–926. 22394517 10.1161/CIRCRESAHA.112.266510 PMC3355870

[36] Wheway G, Nazlamova L, Hancock JT. Signaling through the primary cilium. Front Cell Dev Biol 2018;6:8. 29473038 10.3389/fcell.2018.00008 PMC5809511

[37] Niehrs C, Da Silva F, Seidl C. Cilia as Wnt signaling organelles. Trends Cell Biol 2025;35:24–32. 38697898 10.1016/j.tcb.2024.04.001

[38] High FA, Jain R, Stoller JZ, Antonucci NB, Lu MM, Loomes KM, Kaestner KH, Pear WS, Epstein JA. Murine Jagged1/Notch signaling in the second heart field orchestrates Fgf8 expression and tissue-tissue interactions during outflow tract development. J Clin Invest 2009;119:1986–1996. 19509466 10.1172/JCI38922 PMC2701882

[39] Luna-Zurita L, Flores-Garza BG, Grivas D, De La Pompa JL. A cooperative response to endocardial NOTCH signaling stimulation regulates transcriptional activity during cardiac valve development and disease running title: NOTCH in cardiac valve transcriptional activity. Circ Res 2023;133:1022–1039. 37961886 10.1161/CIRCRESAHA.123.323474 PMC10699509

[40] MacGrogan D, Luxán G, de la Pompa JL. Genetic and functional genomics approaches targeting the Notch pathway in cardiac development and congenital heart disease. Brief Funct Genomics 2014;13:15–27. 24106100 10.1093/bfgp/elt036

[41] Henderson DJ, Eley L, Chaudhry B. New concepts in the development and malformation of the arterial valves. J Cardiovasc Dev Dis 2020;7:38. 32987700 10.3390/jcdd7040038 PMC7712390

[42] Soto-Navarrete MT, López-Unzu MÁ, Durán AC, Fernández B. Embryonic development of bicuspid aortic valves. Prog Cardiovasc Dis 2020;63:407–418. 32592706 10.1016/j.pcad.2020.06.008

[43] Hurwitz LE, Roberts WC. Quadricuspid semilunar valve. Am J Cardiol 1973;31:623–626. 4698133 10.1016/0002-9149(73)90332-9

[44] Fernández B, Fernández MC, Durán AC, López D, Martire A, Sans-Coma V. Anatomy and formation of congenital bicuspid and quadricuspid pulmonary valves in Syrian hamsters. Anat Rec 1998;250:70–79. 9458068 10.1002/(SICI)1097-0185(199801)250:1<70::AID-AR7>3.0.CO;2-I

[45] Nomura-Kitabayashi A, Phoon CKL, Kishigami S, Rosenthal J, Yamauchi Y, Abe K, Yamamura K-I, Samtani R, Lo CW, Mishina Y. Outflow tract cushions perform a critical valve-like function in the early embryonic heart requiring BMPRIA-mediated signaling in cardiac neural crest. Am J Physiol Heart Circ Physiol 2009;297:1617–1628. 10.1152/ajpheart.00304.2009 PMC2781366 19717734

[46] Nakamura E, Makita Y, Okamoto T, Nagaya K, Hayashi T, Sugimoto M, Manabe H, Taketazu G, Kajino H, Fujieda K. 5.78 Mb terminal deletion of chromosome 15q in a girl, evaluation of NR2F2 as candidate gene for congenital heart defects. Eur J Med Genet 2011;54:354–356. 21172461 10.1016/j.ejmg.2010.12.004

[47] Nakamura Y, Taniguchi I, Saiki M, Morimoto K, Yamaga T. Quadricuspid aortic valve associated with aortic stenosis and regurgitation. Jpn J Thorac Cardiovasc Surg 2001;49:714–716. 11808094 10.1007/BF02913511

[48] Yuan SM . Quadricuspid aortic valve: a comprehensive review. Braz J Cardiovasc Surg 2016;31:454–460. 28076624 10.5935/1678-9741.20160090 PMC5407143

[49] Ingham PW . Hedgehog signaling. Curr Top Dev Biol 2022;149:1–58. 35606054 10.1016/bs.ctdb.2022.04.003

[50] Smoak IW, Byrd NA, Abu-Issa R, Goddeeris MM, Anderson R, Morris J, Yamamura K, Klingensmith J, Meyers EN. Sonic hedgehog is required for cardiac outflow tract and neural crest cell development. Dev Biol 2005;283:357–372. 15936751 10.1016/j.ydbio.2005.04.029

[51] Alfieri CM, Cheek J, Chakraborty S, Yutzey KE. Wnt signaling in heart valve development and osteogenic gene induction. Dev Biol 2010;338:127–135. 19961844 10.1016/j.ydbio.2009.11.030 PMC2814915

[52] Pala R, Alomari N, Nauli SM. Primary cilium-dependent signaling mechanisms. Int J Mol Sci 2017;18:2272. 29143784 10.3390/ijms18112272 PMC5713242

[53] Yanardag S, Pugacheva EN. Primary cilium is involved in stem cell differentiation and renewal through the regulation of multiple signaling pathways. Cells 2021;10:1428. 34201019 10.3390/cells10061428 PMC8226522

[54] Ezratty EJ, Pasolli HA, Fuchs E. A presenilin-2–ARF4 trafficking axis modulates Notch signaling during epidermal differentiation. J Cell Biol 2016;214:89–101. 27354375 10.1083/jcb.201508082 PMC4932368

[55] Kong JH, Yang L, Dessaud E, Chuang K, Moore DM, Rohatgi R, Briscoe J, Novitch BG. Notch activity modulates the responsiveness of neural progenitors to sonic hedgehog signaling. Dev Cell 2015;33:373–387. 25936505 10.1016/j.devcel.2015.03.005 PMC4449290

[56] MacGrogan D, D’Amato G, Travisano S, Martinez-Poveda B, Luxán G, del Monte-Nieto G, Papoutsi T, Sbroggio M, Bou V, Gomez-del Arco P, Gómez MJ, Zhou B, Redondo JM, Jiménez-Borreguero LJ, de la Pompa JL. Sequential ligand-dependent notch signaling activation regulates valve primordium formation and morphogenesis. Circ Res 2016;118:1480–1497. 27056911 10.1161/CIRCRESAHA.115.308077

[57] Rochais F, Kelly RG, Rammah M, Théveniau-Ruissy M, Sturny R. PPARγ and NOTCH regulate regional identity in the murine cardiac outflow tract. Circ Res 2022;131:842–858. 36205127 10.1161/CIRCRESAHA.122.320766

[58] Seya D, Ihara D, Shirai M, Kawamura T, Watanabe Y, Nakagawa O. A role of Hey2 transcription factor for right ventricle development through regulation of Tbx2-Mycn pathway during cardiac morphogenesis. Dev Growth Differ 2021;63:82–92. 33410138 10.1111/dgd.12707

[59] Van Dam TJP, Kennedy J, van der Lee R, de Vrieze E, Wunderlich KA, Rix S, Dougherty GW, Lambacher NJ, Li C, Jensen VL, Leroux MR, Hjeij R, Horn N, Texier Y, Wissinger Y, van Reeuwijk J, Wheway G, Knapp B, Scheel JF, Franco B, Mans DA, van Wijk E, Képès F, Slaats GG, Toedt G, Kremer H, Omran H, Szymanska K, Koutroumpas K, Ueffing M, Nguyen T-MT, Letteboer SJF, Oud MM, van Beersum SEC, Schmidts M, Beales PL, Lu Q, Giles RH, Szklarczyk R, Russell RB, Gibson TJ, Johnson CA, Blacque OE, Wolfrum U, Boldt K, Roepman R, Hernandez-Hernandez V, Huynen MA. CiliaCarta: an integrated and validated compendium of ciliary genes. PLoS One 2019;14:e0216705. 31095607 10.1371/journal.pone.0216705 PMC6522010

[60] Calvo SE, Clauser KR, Mootha VK. MitoCarta2.0: an updated inventory of mammalian mitochondrial proteins. Nucleic Acids Res 2016;44:D1251–D1257. 26450961 10.1093/nar/gkv1003 PMC4702768

