# Supplementary material for: Remodelling of supernumerary leaflet primordia leads to bicuspid aortic valve caused by loss of primary cilia
Source: Cardiovasc Res. 2025 Jun 11;121(11):1750–66. doi: 10.1093/cvr/cvaf108 (PMC12477680; doi:10.1093/cvr/cvaf108)

Supplementary Figure 1

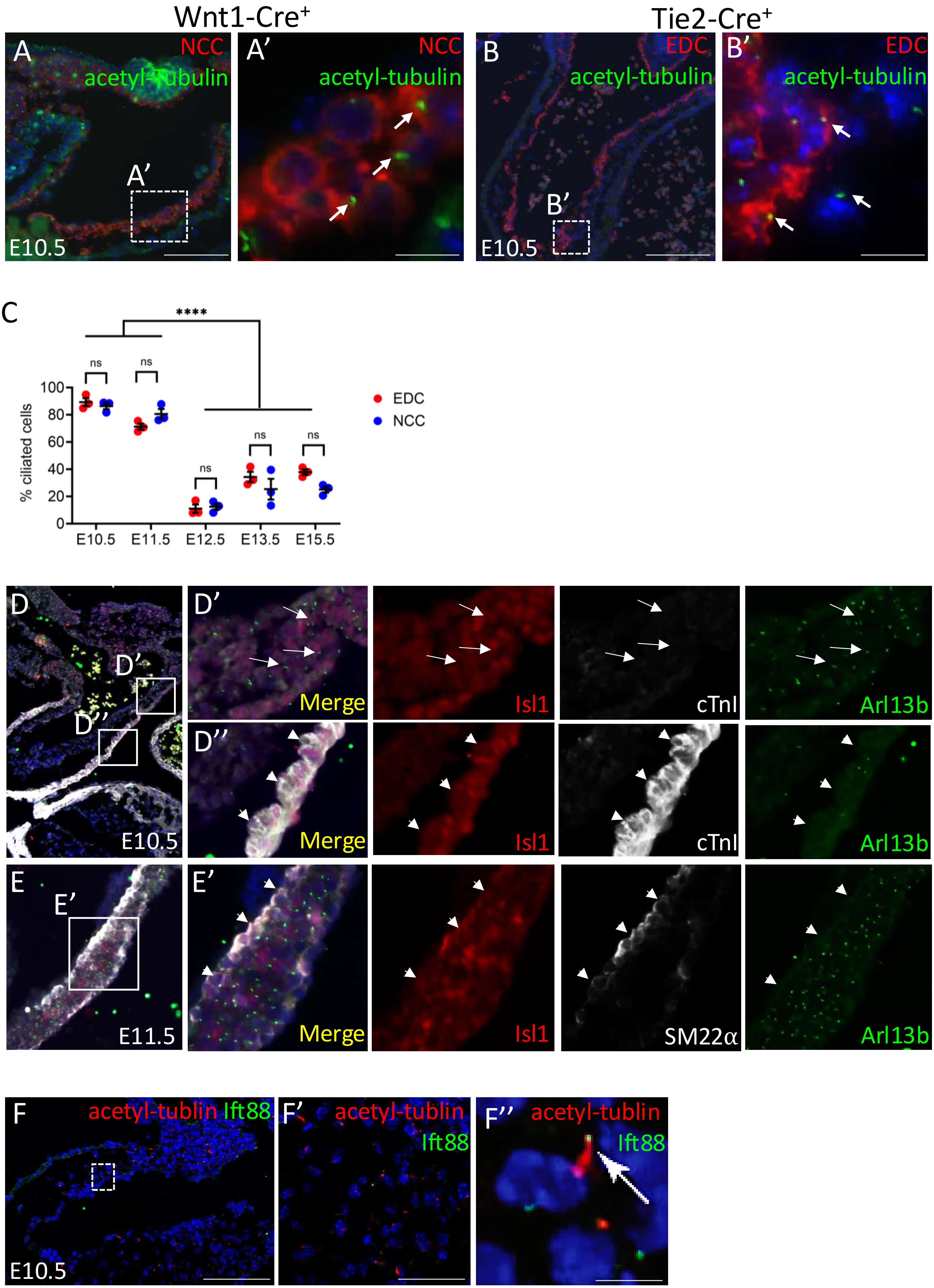

Supplementary Figure 2

A

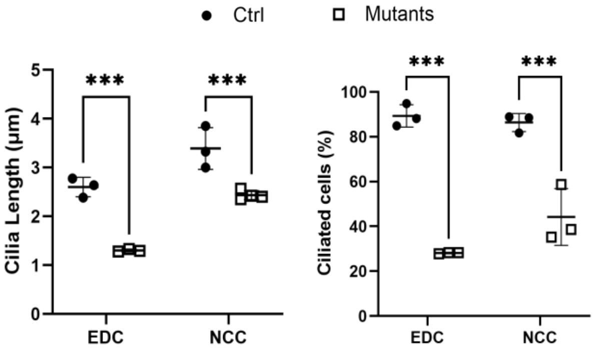

B

	Mutant	Total	Survival %	Expected %
Ift88$^{f/+}$; Wnt1-cre	3	33	8.6%*	12.5%
Ift88$^{f/f}$; Wnt1-Cre	5	27	18.5%**	25%
Ift88$^{f/f}$; Tie2-Cre	20	69	29%	25%
Ift88$^{f/f}$; Tnnt2-Cre	9	60	21%	25%

Chi squared equal 0.390* and 0.605**

C

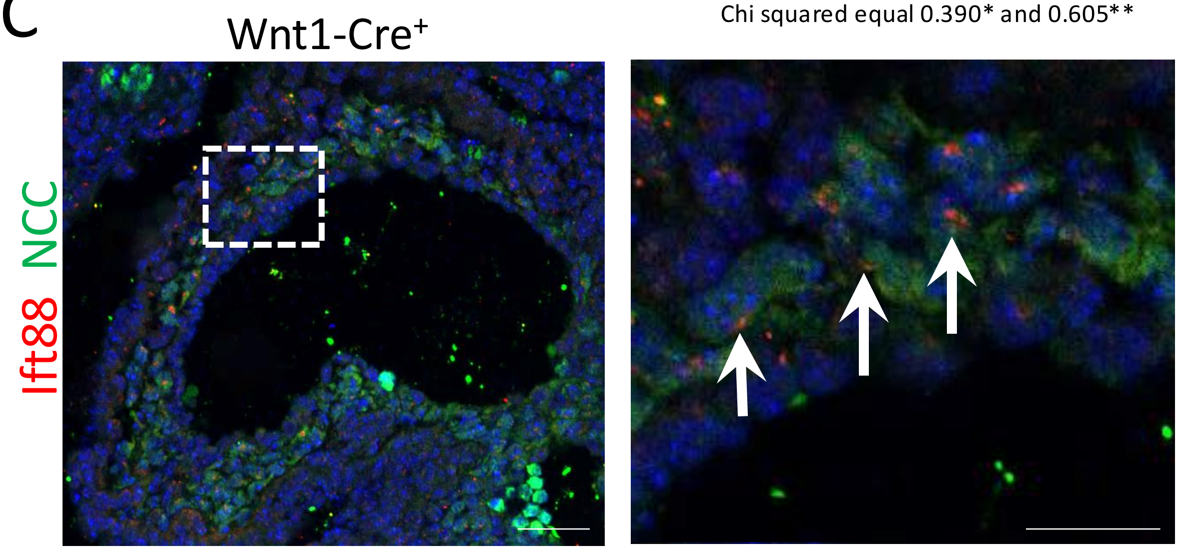

D

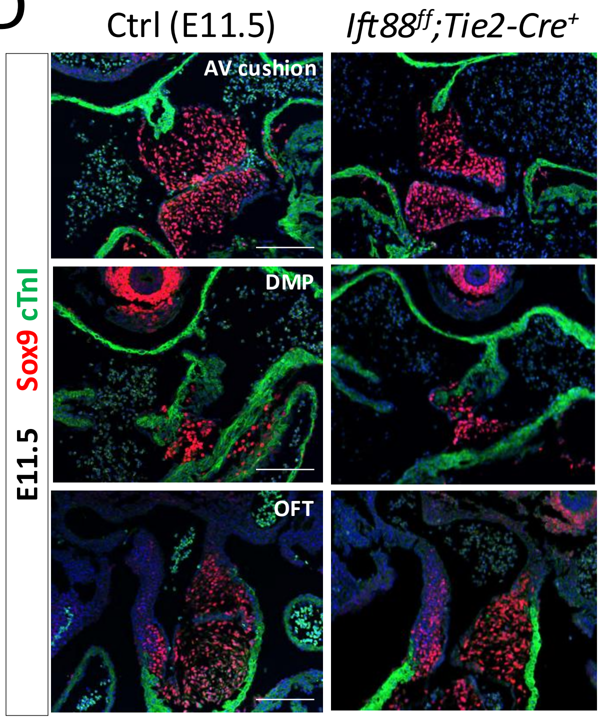

Supplementary Figure 3

A

		Ift88;TnnTCre (n=8)				
		cAVSD	mVSD	membVSD	PPAT	Arterial valve defects
	% of overall phenotypes	0%	25%	12.5%	12.50%	25%
Fetus code	1		✓	✓		
	2		✓			
	3				✓	
	4					✓
	5					✓
	6					
	7					
	8					

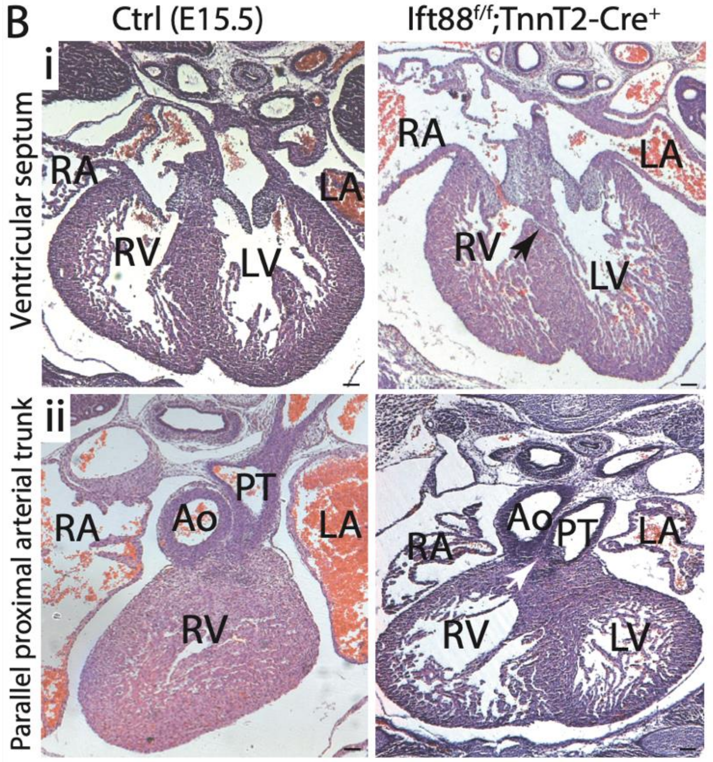

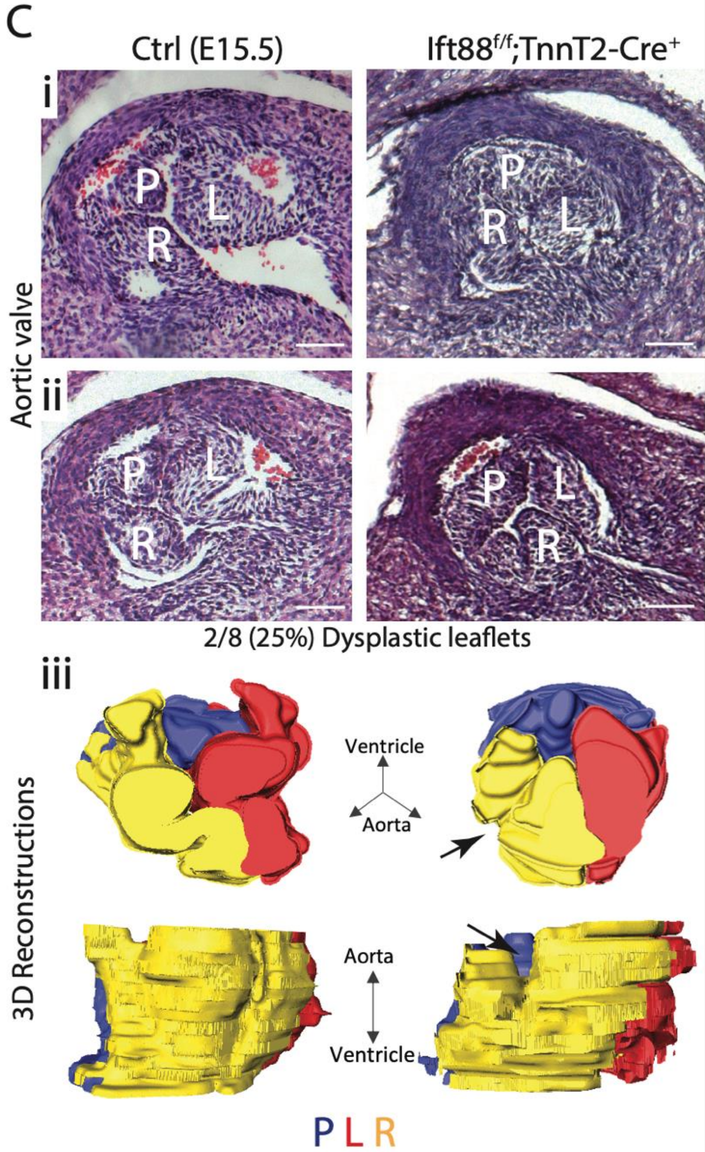

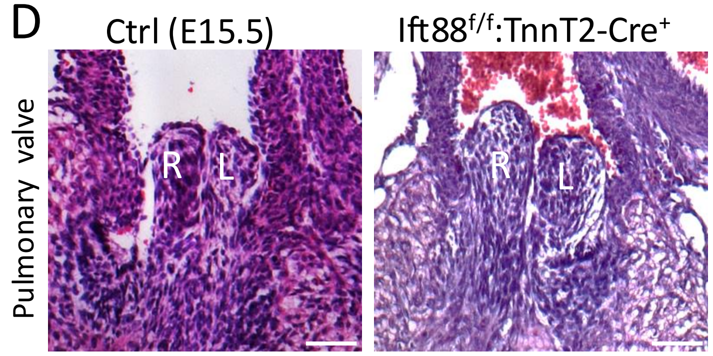

Supplementary Figure 4

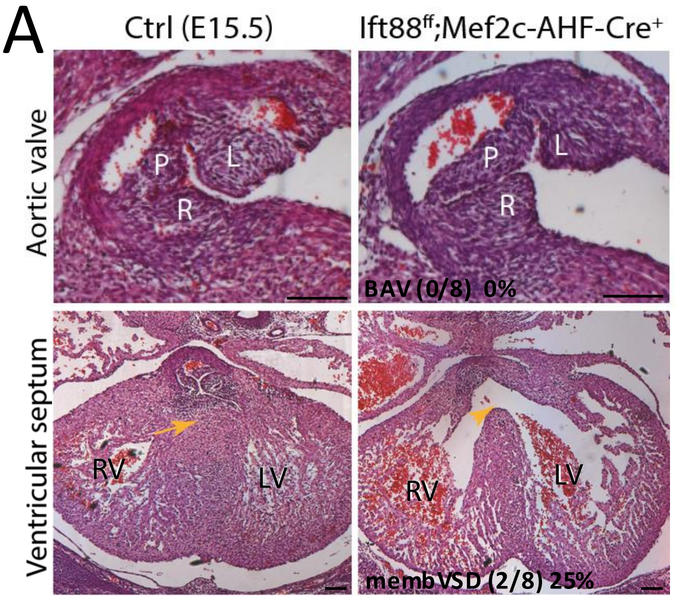

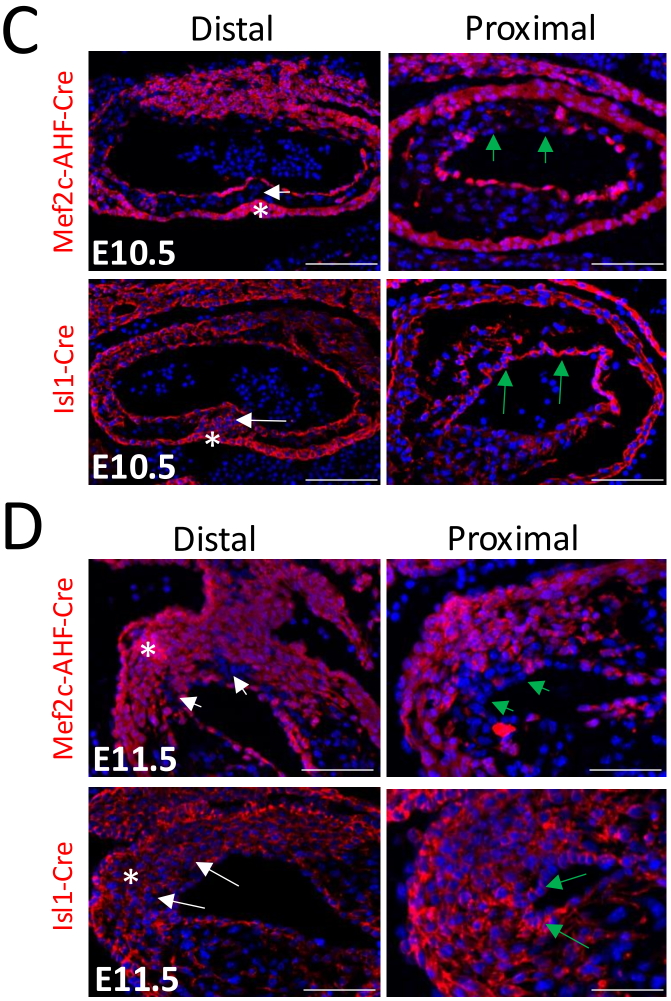

B

	Mutant	Total	Survival %	Expected %
$Ift88^{f/f}$; Mef2c-AHF-Cre	11	34	32%*	25%
$Ift88^{f/f}$; Isl1-Cre	19	71	27%	25%

*Chi squared equals 0.980

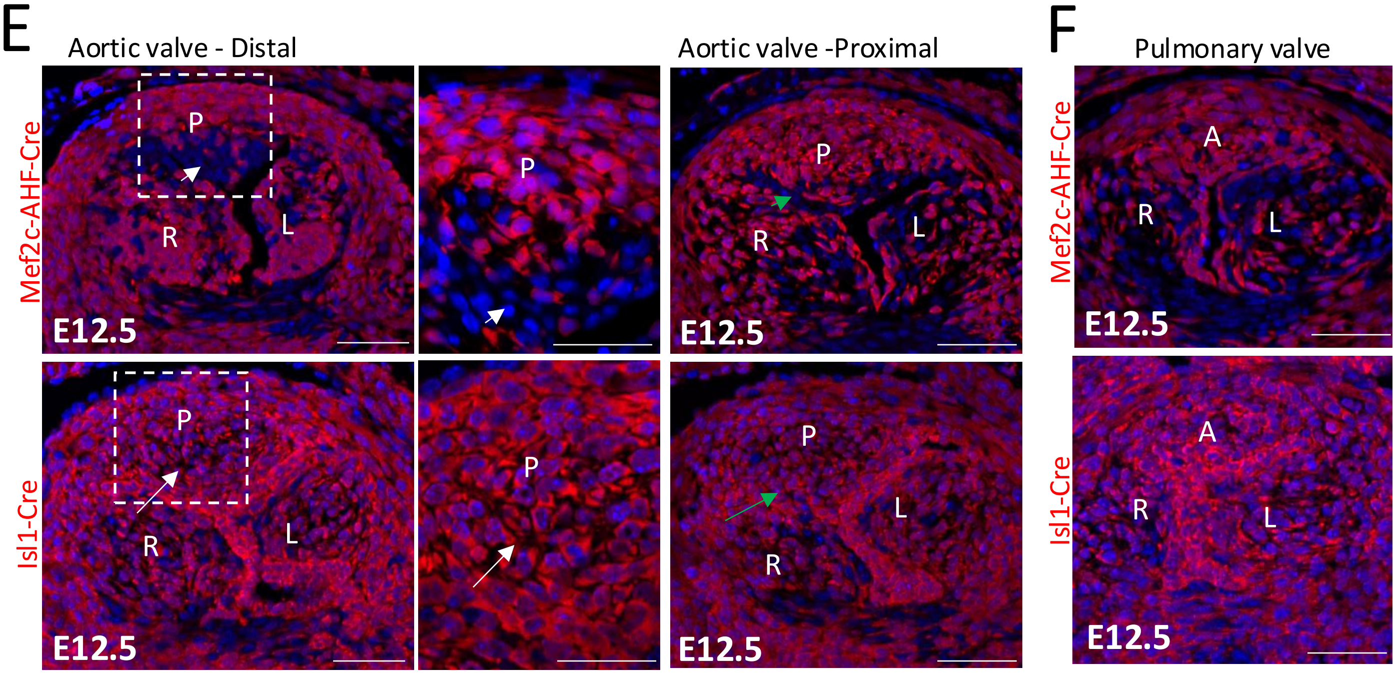

Supplementary Figure 5

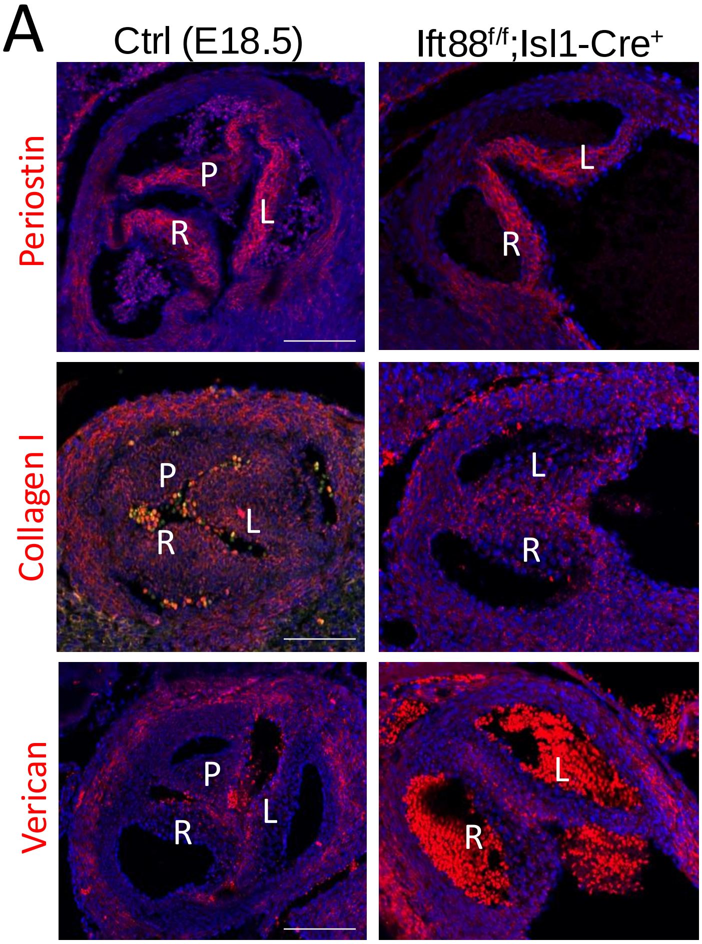

Supplementary Figure 6

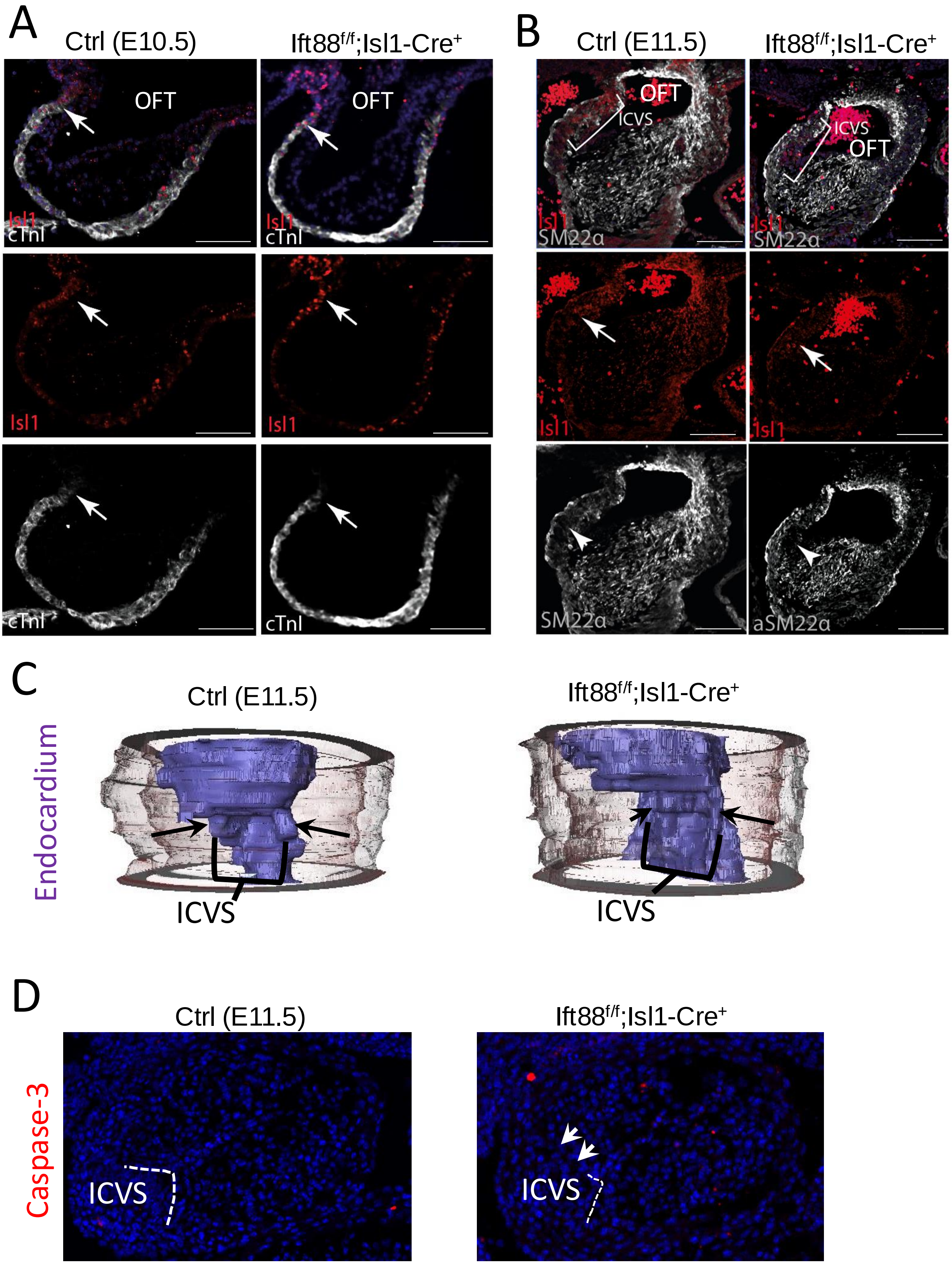

Supplementary Figure 7

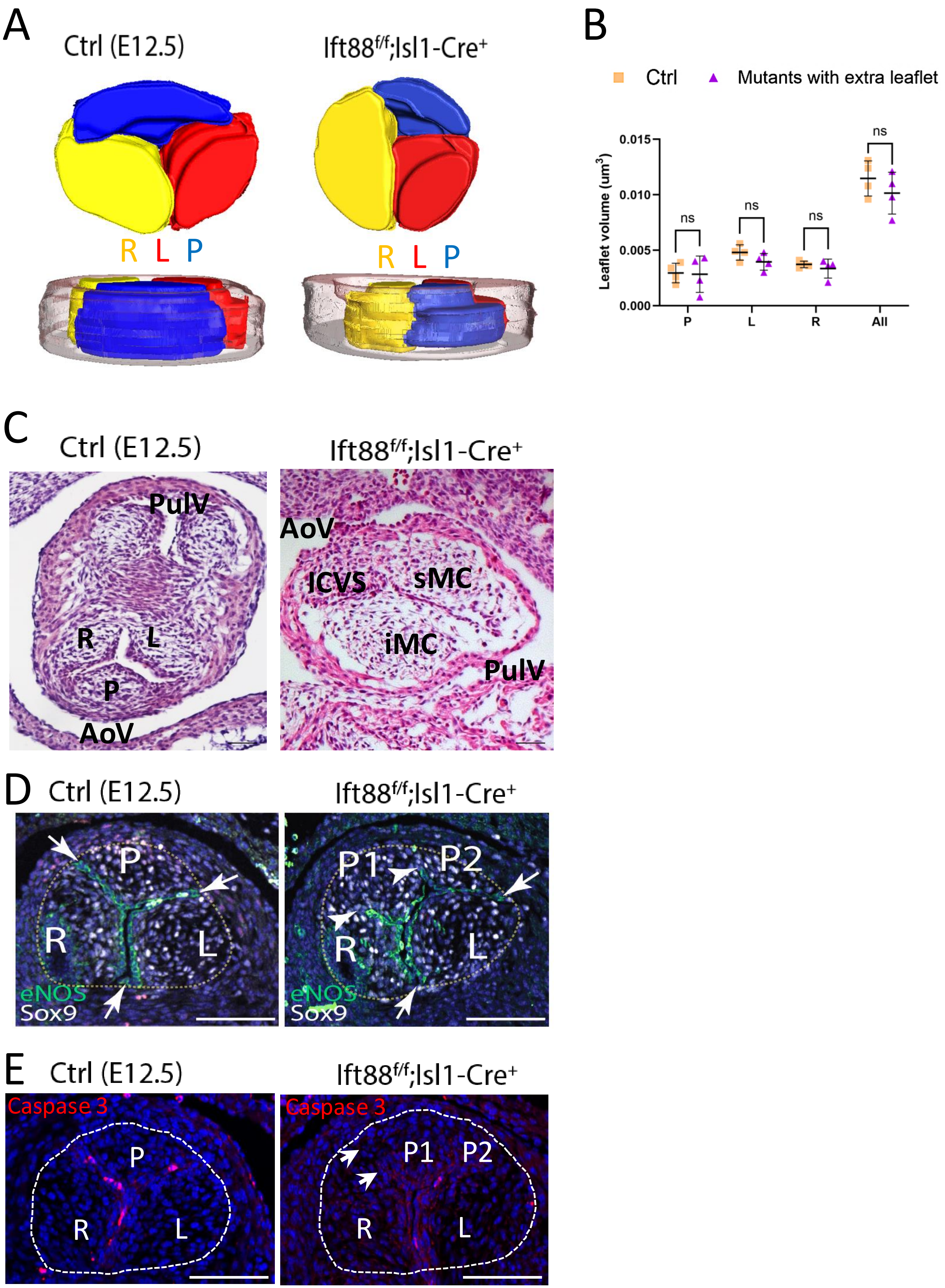

Supplementary Figure 8

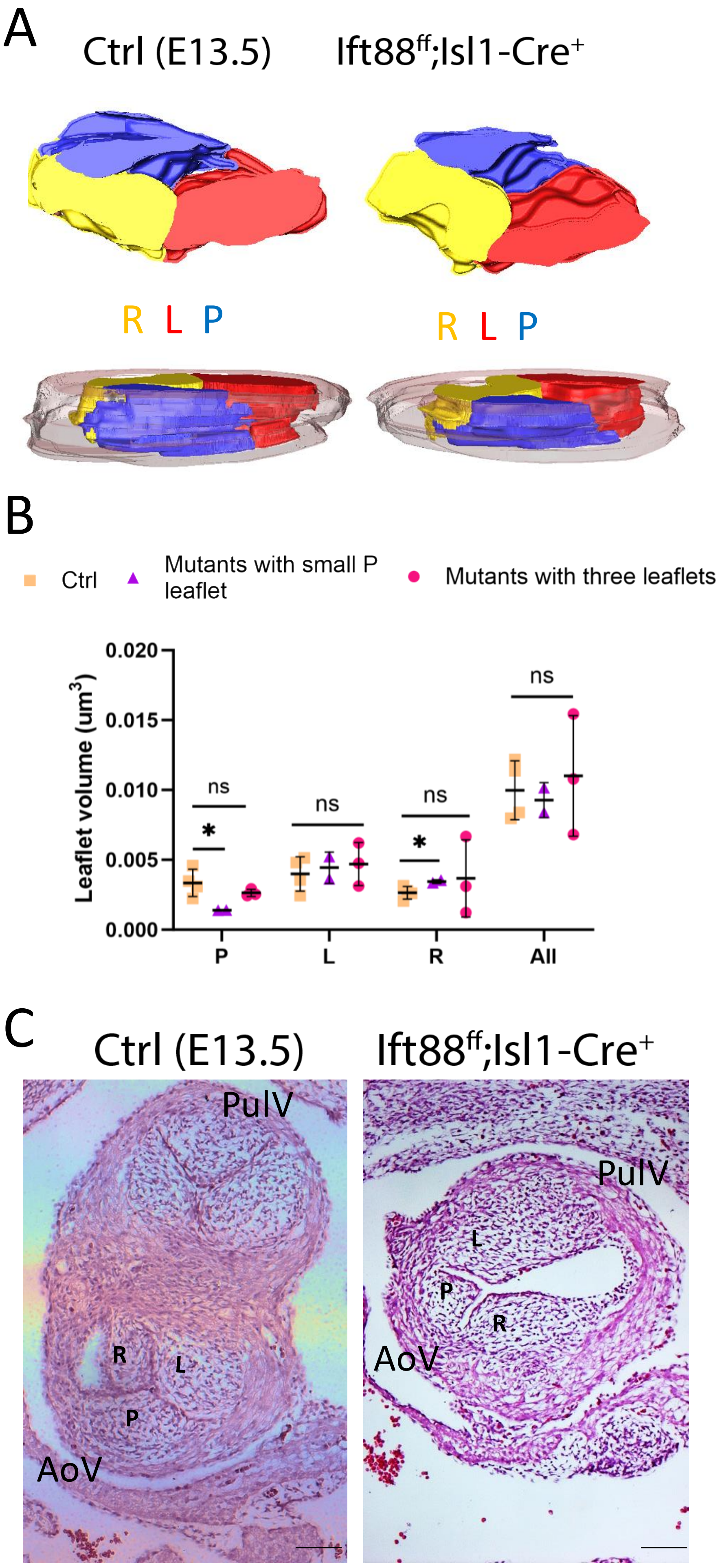

Supplementary Figure 9

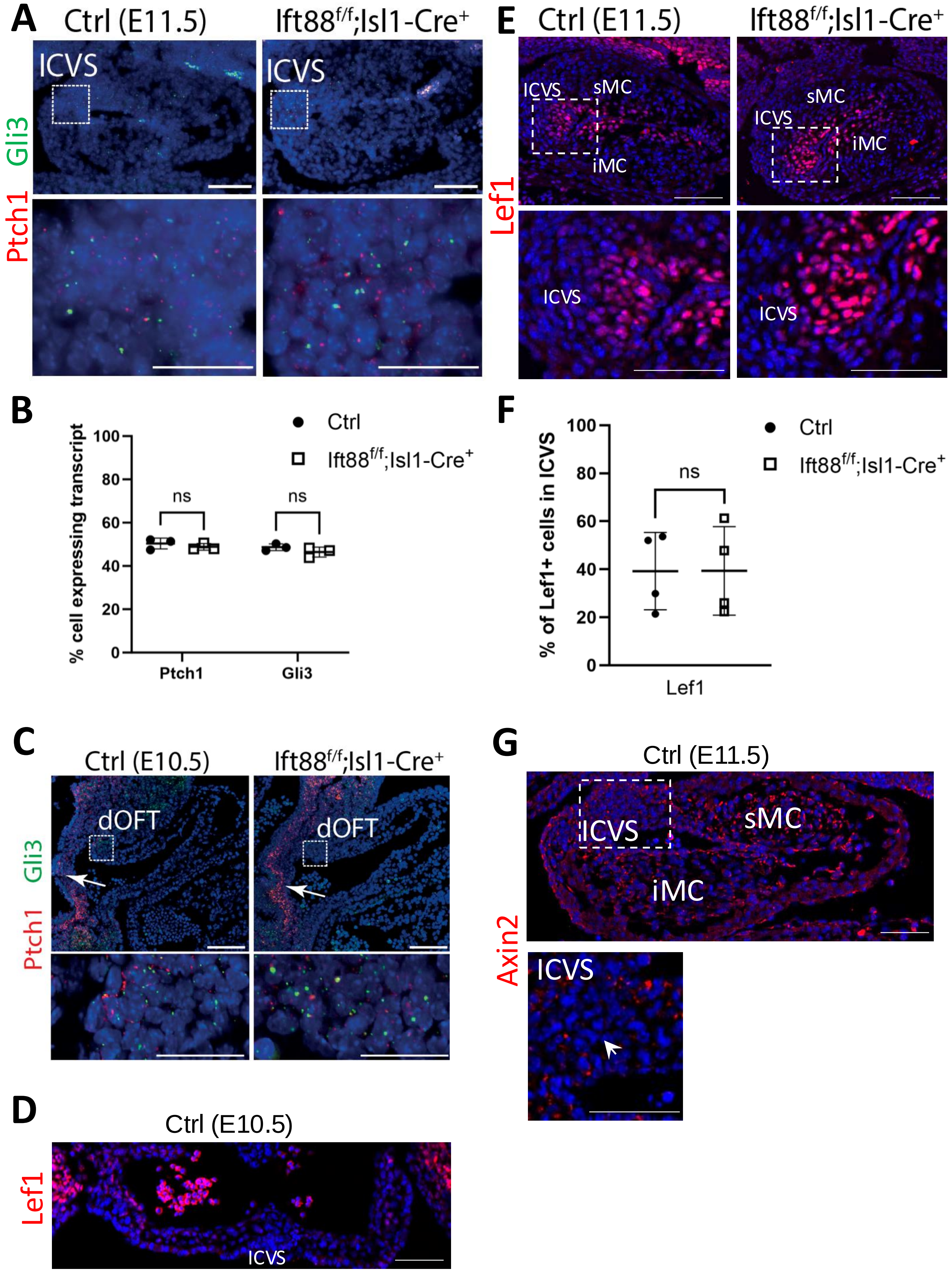

Supplementary Figure 10

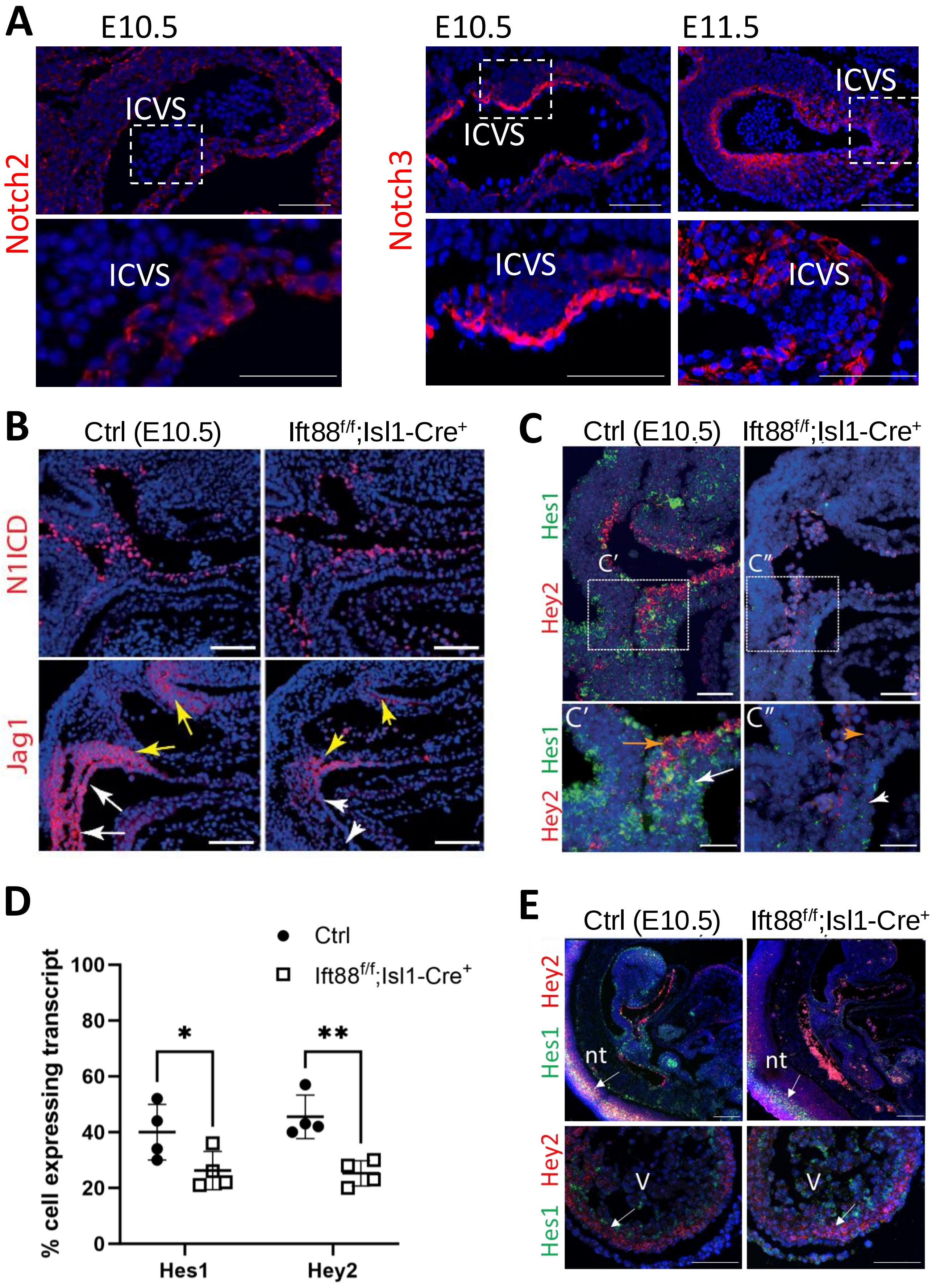

Supplementary Figure 11

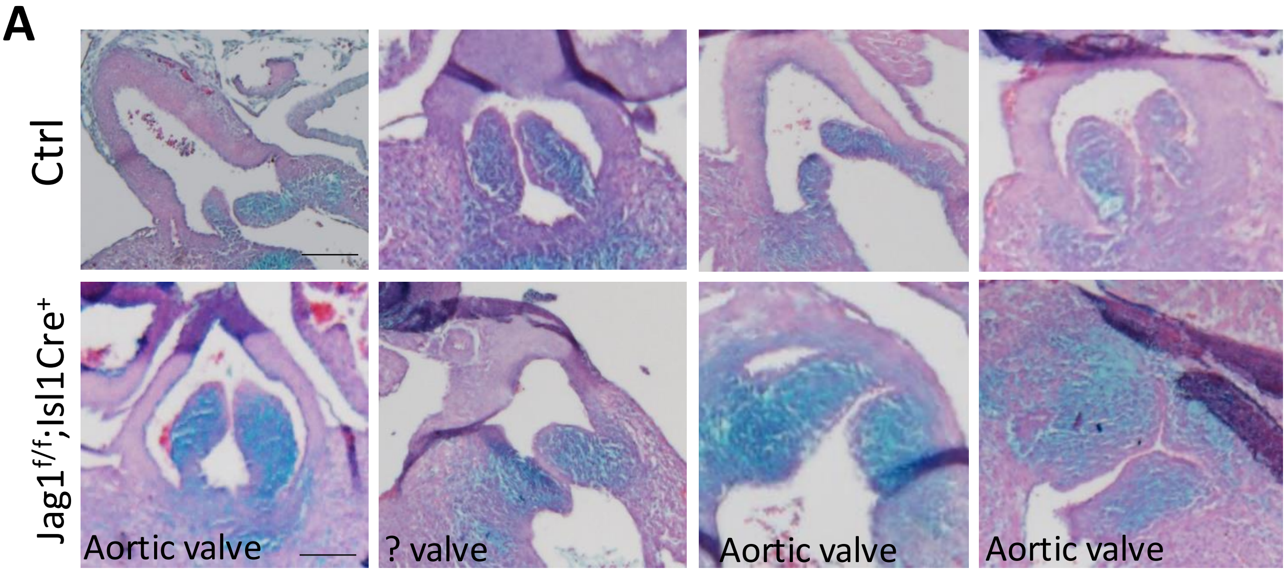

Supplement: cvaf108_Supplementary_Data [file cvaf108_supplementary_data.zip › Suppl Figure 14.03.25.pdf]
